# Using online tools at the Bovine Genome Database to manually annotate genes in the new reference genome

**DOI:** 10.1111/age.12962

**Published:** 2020-06-14

**Authors:** D. A. Triant, J. J. Le Tourneau, C. M. Diesh, D. R. Unni, M. Shamimuzzaman, A. T. Walsh, J. Gardiner, A. K. Goldkamp, Y. Li, H. N. Nguyen, C. Roberts, Z. Zhao, L. J. Alexander, J. E. Decker, R. D. Schnabel, S. G. Schroeder, T. S. Sonstegard, J. F. Taylor, R. M. Rivera, D. E. Hagen, C. G. Elsik

**Affiliations:** ^1^ Division of Animal Sciences University of Missouri Columbia MO 65211 USA; ^2^ Department of Bioengineering University of California, Berkeley Berkeley CA 94720 USA; ^3^ Division of Environmental Genomics and Systems Biology Lawrence Berkeley National Laboratory Berkeley CA 94608 USA; ^4^ Department of Animal and Food Sciences Oklahoma State University Stillwater OK 74078 USA; ^5^ MU Institute for Data Science and Informatics University of Missouri Columbia MO 65211 USA; ^6^ Division of Plant Sciences University of Missouri Columbia MO 65211 USA; ^7^ USDA‐ARS‐PA‐Livestock & Range Research Lab Miles City MT 59301 USA; ^8^ USDA‐ARS Animal Genomics and Improvement Lab Beltsville MD 20705 USA; ^9^ Acceligen Eagan MN 55121 USA

**Keywords:** *Bos taurus*, gene prediction, genome annotation, genome annotation tools, RNA‐seq

## Abstract

With the availability of a new highly contiguous *Bos taurus* reference genome assembly (ARS‐UCD1.2), it is the opportune time to upgrade the bovine gene set by seeking input from researchers. Furthermore, advances in graphical genome annotation tools now make it possible for researchers to leverage sequence data generated with the latest technologies to collaboratively curate genes. For many years the Bovine Genome Database (BGD) has provided tools such as the apollo genome annotation editor to support manual bovine gene curation. The goal of this paper is to explain the reasoning behind the decisions made in the manual gene curation process while providing examples using the existing BGD tools. We will describe the sources of gene annotation evidence provided at the BGD, including RNA‐seq and Iso‐Seq data. We will also explain how to interpret various data visualizations when curating gene models, and will demonstrate the value of manual gene annotation. The process described here can be applied to manual gene curation for other species with similar tools. With a better understanding of manual gene annotation, researchers will be encouraged to edit gene models and contribute to the enhancement of livestock gene sets.

## Introduction

Livestock productivity has increased with technological advances, including those that incorporate genetic modifications (Thornton 2010). The emergence of genomic resources has helped to identify variants creating differences in agriculturally important traits, which can improve production efficiency, helping to balance demand against environmental impact (Georges *et al*. 2019). The bovine (*Bos taurus*) genome has been used to identify genes associated with such complex traits (e.g. Meredith *et al*. 2012; Thompson‐Crispi *et al*. 2014). High‐quality genome annotation is essential for understanding biological mechanisms and targeting genes of interest. Assembled genomes are typically annotated using computational methods, and the resulting gene sets are then further refined by manual curation. Livestock sequencing projects usually do not have sufficient resources to employ biocurators to manually inspect and correct all predicted gene sequences. However, collaborative manual annotation efforts by scientists with diverse areas of expertise can enhance the quality of livestock gene sets.

An early assembly of the bovine genome was annotated and analyzed by The Bovine Genome Sequencing and Analysis Consortium, which at the time was the largest group annotation project conducted for any mammalian genome (Bovine Genome Sequencing & Analysis Consortium 2009). The Bovine Genome Database (BGD; http://bovinegenome.org; Shamimuzzaman *et al*. 2020) supported the Consortium with genome annotation tools, and since then has provided genome browsers for various bovine genome releases. The BGD currently provides the latest annotation tools (Shamimuzzaman *et al*. 2020) for the new highly contiguous bovine reference genome assembly, ARS‐UCD1.2 (Rosen *et al*. 2020). The BGD uses apollo2 (Dunn *et al*. 2019), a plugin for the jbrowse genome browser (Buels *et al*. 2016). With manual genome annotation performed in a web browser, edits are instantly saved, made available for viewing by others and recorded into a history that is available for inspection.

Whereas the bovine reference genome assembly has been considerably upgraded, challenges in automated gene prediction can still lead to erroneous models or missing genes. Examples of how researchers can refine bovine predicted genes using tools at the BGD include: resolution of disagreements between RefSeq and Ensembl gene models; the addition of novel genes and transcript isoforms; the extension of partial coding exons or UTRs; and the correction of exons that are incongruent with transcriptome evidence. The annotation tools at the BGD serve not only those who wish to improve the catalog of bovine genes, but also those who wish to verify genes of interest in their research, particularly when an analysis using predicted gene sets such as RefSeq and Ensembl leads to unexpected results. For example, an analysis to detect variants in RNA‐seq data may reveal variants within introns, but manual gene annotation may reveal that the variants are located in exons of previously unannotated isoforms.

Whereas reports have described gene annotation software and the biological rationale for manual gene annotation (e.g. Loveland *et al*. 2012; Dunn *et al*. 2019), there is little available literature describing the reasoning applied *during* the manual annotation process. Based on our previous experience (Reese *et al*. 2010), researchers are often hesitant to edit gene models because they are concerned that they will make poor annotation decisions. Yet investigators often identify problematic gene models in the process of their own research. With access to annotation tools and data at the BGD, users can evaluate and, if necessary, correct problematic gene models that directly impact their research, while contributing gene model curations that benefit the entire research community. The objectives of this article are to describe the sources of bovine gene annotation evidence provided at the BGD and explain how to interpret the evidence tracks when modifying a gene model. Examples using BGD tools and data will illustrate both the gene curation process and the benefits of manual gene annotation. More than a technical protocol, this article demonstrates the thought process involved in gene model curation. By explaining the reasoning behind decisions made in the manual gene annotation process, we will instill confidence and encourage researchers to contribute to the improvement of the bovine gene set, and further inspire manual gene curation efforts for additional livestock species.

## Materials and methods

### Genome and gene sets

The bovine ARS‐UCD1.2 genome assembly and RefSeq gene set were downloaded from NCBI (ftp://ftp.ncbi.nlm.nih.gov/genomes/refseq/vertebrate_mammalian/Bos_taurus/latest_assembly_versions/GCF_002263795.1_ARS‐UCD1.2/). The Ensembl gene set (Ensembl95 Release) was downloaded from Ensembl (http://ftp.ensembl.org/pub/release‐95/gff3/bos_taurus/).

### Transcriptome data

#### Transcriptome data sources

Transcriptome tracks were created using single‐end (SE) RNA‐seq, paired‐end (PE) RNA‐seq and Iso‐Seq data, predominantly from tissues isolated from the reference genome individual (Line 1 Hereford Dominette 01449) and closely related individuals (Table S1). Samples for SE RNA‐seq included 79 tissues from Dominette, two tissues from Dominette’s female calf, three tissues from Dominette’s male fetus and 10 tissues from Dominette’s sire, Domino 99247. mRNA libraries were prepared using the Illumina TruSeq RNA Sample Preparation Kit and sequenced on the Illumina HiSeq 2000 to generate 100 bp SE reads. Libraries for four pairs of tissue samples with identical barcodes were mistakenly pooled prior to sequencing, resulting in 90 NCBI BioSamples and 90 SRA experiment accessions (Table S1). Later, mRNA libraries from 16 of the same Dominette tissues were prepared using the Illumina TruSeq Stranded RNA Sample Preparation Kit and sequenced on Illumina HiSeq 2000 to generate 100 bp PE reads. Transcriptome data at the BGD also include data reported in Rosen *et al*. (2020): PE RNA‐seq from 24 tissues from Dominette and her sire (BioProject PRJNA379574), as well as Iso‐Seq from 22 tissues of Dominette and her sire (BioProject PRJNA386670) and the lactating mammary gland of a Holstein Friesian individual (BioProject PRJNA434299).

#### Creating RNA‐Seq tracks

Fastq‐formatted RNA‐seq sequences were adapter trimmed using fastq‐mcf (https://code.google.com/p/ea‐utils/wiki/FastqMcf), quality trimmed using dynamictrim (Cox *et al*. 2010) and quality checked using fastqc (http://www.bioinformatics.babraham.ac.uk/projects/fastqc/). The trimmed reads were aligned to the unmasked ARS‐UCD1.2 genome assembly with Hisat2 (Kim *et al*. 2015), using command line options appropriate for either SE or stranded PE reads. We used stringtie (Pertea *et al*. 2015) to assemble RNA‐seq read alignments into transcripts using the ‐rf option for the stranded PE reads and not using that option for the non‐stranded SE reads.

#### Creating Iso‐Seq tracks

**Figure 1:**
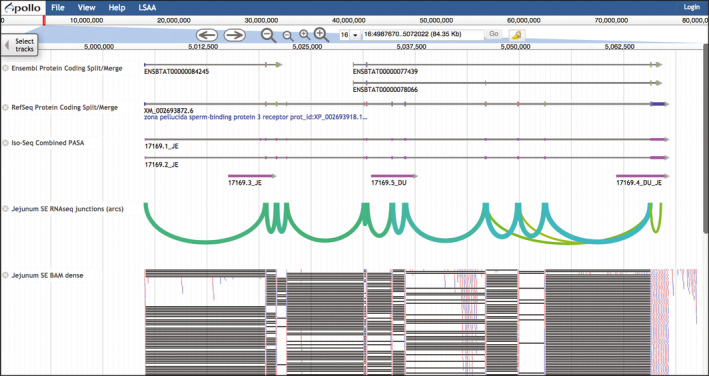
Bovine Genome Database (BGD) jbrowse. This view of the BGD jbrowse genome browser shows an example of a split/merge disagreement between Ensembl and RefSeq genes. Ensembl shows two genes, one of which has two transcripts, where RefSeq shows one gene. The ‘Iso‐Seq Combined PASA’ track includes two transcripts that are similar to the RefSeq transcript, with identifiers that include the two‐letter code ‘JE’ for jejunum. The green arcs in the ‘Jejunum SE RNAseq junctions’ track highlight RNA‐seq splice junctions. The ‘Jejunum SE BAM dense track’ shows RNA‐seq read alignments as small red or blue bars and connections between parts of spliced reads as gray lines. Both the Iso‐Seq and the RNA‐seq data support the RefSeq transcript or a merge of the Ensembl gene models into one gene.

Unmasked polished Iso‐Seq reads were aligned to the unmasked ARS‐UCD1.2 assembly using gmap (Wu & Watanabe 2005) with the options: ‐f 3 ‐n 0 ‐x 50 ‐t 6 ‐B 5 ‐z sense_filter. The results of the gmap alignments are provided as genome browser tracks called ‘Iso‐Seq gmap Alignment’ in BGD jbrowse. gmap alignments with at least 95% identity, covering at least 75% of the Iso‐Seq read length, were then used in reference genome‐based transcript assembly with PASA (Haas *et al*. 2003) for each tissue separately using the ‐‐transcribed_is_aligned_orient flag. The outputs of this initial PASA assembly step are provided as individual genome browser tracks for each tissue called ‘Iso‐Seq PASA Assembly’ in BGD jbrowse. Resulting PASA transcript assemblies for each tissue were then assigned unique identifiers that included a tissue identifier, and loaded into a single new pasa database, so that PASA could be used to create a single combined set of transcript assemblies. Transcripts with identical introns were merged into one representative transcript, extending the 5′ and 3′ ends to the furthest extent of any of the individual component transcripts. We call the transcripts generated in the combination step ‘unique transcripts’. We used a single‐linkage clustering algorithm to group unique transcripts into a single gene locus if they had overlapping exons. The result of the last PASA assembly and clustering step is a single genome browser track called ‘Iso‐Seq Combined PASA Assembly’ (e.g. Fig. 1).

## Results and discussion

Here we demonstrate the thought process and general methods employed when annotating a gene using tools at the BGD, after providing essential information for accessing BGD apollo and selecting gene evidence tracks. Specific bovine gene annotation examples provided in Appendices S1 and S2 show the reasoning used in correcting specific gene model problems. Non‐BGD‐specific technical instructions for using apollo are provided in the apollo user documentation available on the apollo website (https://genomearchitect.readthedocs.io/en/latest/UsersGuide.html) and in Dunn *et al*. (2019).

### Registering for BGD apollo

apollo registration is available by clicking the apollo tab in the BGD navigation bar. Registration is a two‐step process, involving both a user submission form, accessed by clicking the ‘Click here to register’ link, and an email response from the BGD administrator. We request users to provide their institutional emails and full names in the registration form. Once the form is submitted, the BGD administrator grants read, write and export access to the user after validating the user’s email address, and then notifies the user that the account has been set up. We take these cautionary measures to protect the submitted data of other users. Furthermore, the use of email addresses as the owner labels for specific gene models facilitates communication between users who share interests in the same genes. A Bovine apollo Demo instance that does not require registration is available via the apollo tab in the BGD navigation bar.

### jbrowse/apollo access, genome navigation and track selection

The annotation tools at the BGD are accessed through the navigation bar at the top of the home page. To access the ARS‐UCD1.2 jbrowse genome browser, click the jbrowse tab and select ARS‐UCD1.2 JBROWSE from the pulldown menu. Without logging in to apollo, jbrowse (Fig. 1) can be used to navigate through the genome, select tracks and view the evidence, but not to view user‐submitted annotations or access the gene editing functions.

#### Information panel

**Figure 2:**
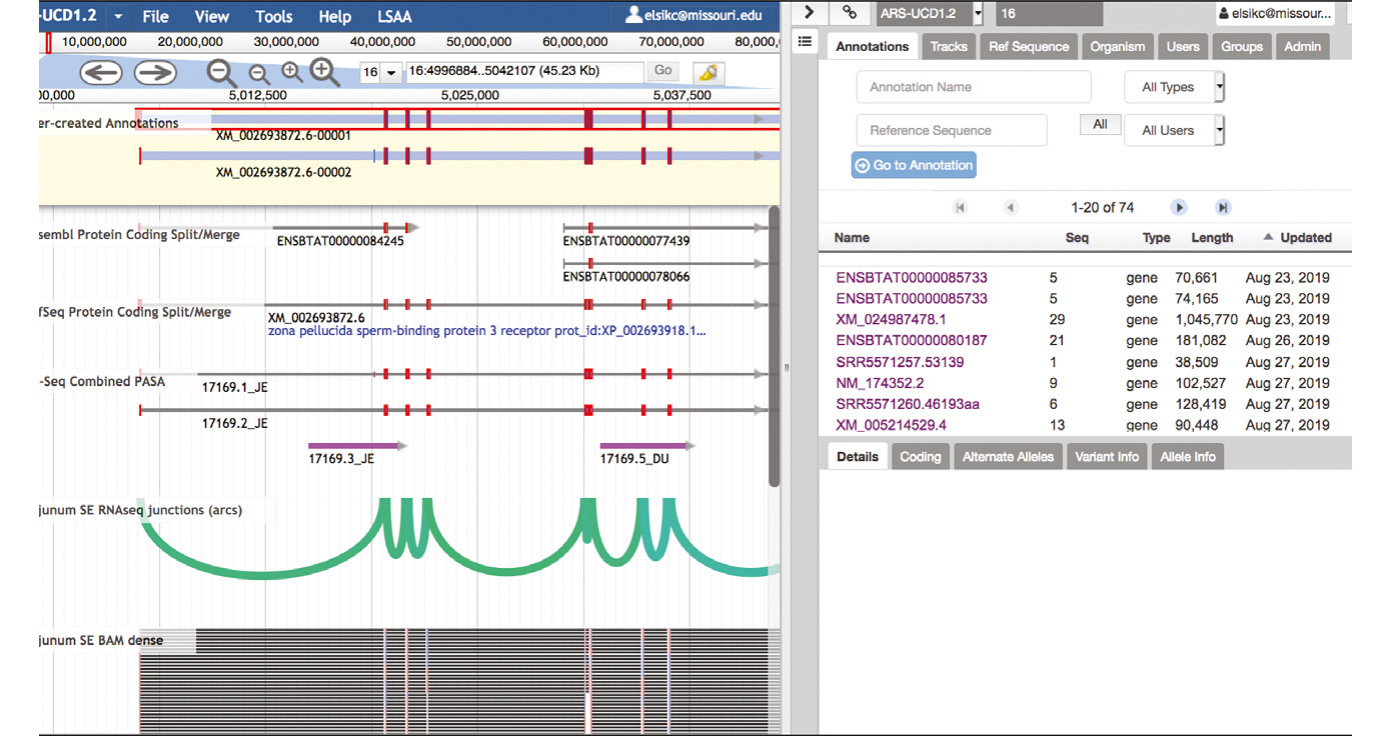
BGD apollo. After logging into apollo, an Information Panel appears on the right. To the left of the Information Panel is the browser, which now includes the Evidence Area (equivalent to the jbrowse view) and the Editing Area, with a light yellow background, above the Evidence Area. Here, the RefSeq transcript and an Iso‐Seq transcript have been dragged to the Editing Area. Notice that the identifiers of the transcripts in the Editing Area both resemble the RefSeq identifier, which was the first transcript added. One transcript has been clicked and is now outlined in red. Exon boundaries in the gene prediction and Iso‐Seq tracks are highlighted in red if they agree with the exon boundaries in the outlined annotation. The ‘Jejunum SE BAM dense’ track has been configured to hide unspliced alignments using a pulldown menu available by clicking the track label. The Information Panel can be hidden from view to increase the browser width by clicking the greater‐than sign near the upper left of the panel. The ‘Select Tracks’ tab seen on the left of the browser in Fig. 1 can be brought back into view by clicking the icon that resembles a list under the greater‐than sign.

After logging in to apollo using the button in the upper‐right corner, the window changes, and is split between the genome browser on the left, which includes the Evidence and Editing Areas, and the Information Panel on the right (Fig. 2). The Information Panel can be hidden by clicking the greater‐than sign at the upper left. The Information Panel provides three functions available with tabs. The Annotation Tab provides a list of submitted annotations, and allows navigation directly to the location of a selected annotation. The Tracks Tab allows the selection of tracks for viewing in the browser. The Ref Sequence Tab provides a list of all available chromosomes and unplaced contigs in the genome assembly.

#### Faceted track selector

The Select Tracks tab that appears in the upper left of a jbrowse window (prior to logging in) allows the Faceted Track Selector to be opened, which provides over 800 tracks organized into categories, with a filterable and searchable table and flexible search functions. Once logged in to apollo, tracks can be selected either with the Tracks Tab in the Information Panel or the Faceted Track Selector. The Select Tracks tab is available by clicking the icon that resembles a list, under the icon used to open or close the Information Panel (described above). On the left of the Faceted Track Selector is a list of tracks organized according to track type, and in reference to gene expression tracks, Organ System and Brenda Tissue Ontology. Highlighting one or more Data Type, Organ System or Brenda Tissue Ontology in the left panel filters the tracks, so that only those tracks are shown in the table on the right. The table, with additional details about the RNA‐seq tracks, is searchable by entering text in the ‘Contains text’ search box above the table. Select tracks for viewing by clicking the boxes on the left side of the table. Clicking ‘Back to Browser’ brings the browser area with selected tracks into view.

### Gene evidence tracks

#### Gene prediction tracks

The types of tracks most often used in manual gene annotation are computed gene prediction and RNA expression tracks. The gene prediction tracks in the BGD represent RefSeq and Ensembl transcripts, including protein‐coding, long non‐coding and other RNA. Gene prediction tracks appear as histograms depicting gene density when zoomed out, and allow the visualization of predicted introns and exons when zoomed in.

#### RNA expression tracks

The BGD provides three kinds of RNA expression data generated from the tissues of Dominette and her close relatives: SE Ilumina RNA‐seq, PE RNA‐seq and Iso‐Seq. Each type of expression data is provided in several visualizations. Iso‐Seq should be the first expression data type investigated, because an Iso‐Seq sequence usually includes the entire transcript, without the need for assembly. In addition to identifying new transcript isoforms, Iso‐Seq can provide evidence to support the merging of gene predictions and to create novel isoform and gene annotations. A disadvantage of the bovine Iso‐Seq data compared with the bovine RNA‐seq data is that there are fewer Iso‐Seq sequenced tissue transcriptomes. Furthermore, the depth of sequencing tends to be lower for Iso‐Seq than RNA‐seq and isoforms found in RNA‐seq data may not be present in the Iso‐Seq data. Of the Iso‐Seq tracks, ‘Iso‐Seq Combined PASA Assembly’ is the track of choice to determine if there is any Iso‐Seq evidence for gene or isoform structure (Fig. 1). The identifier for each feature indicates the tissues that the transcript was found in, using two‐letter abbreviations (Table S2). Once tissues have been identified, they can be further investigated using the tissue‐specific Iso‐Seq tracks. Of these, the Iso‐Seq gmap tracks provide the most reliable information, because the transcripts have not been further assembled, which could introduce errors. On the other hand, some large genes, such as titin (*TTN*) are prone to produce fragments even with Iso‐Seq, and assembly with PASA can help reconstruct full‐length isoforms, as well as reduce redundancy.

The bovine RNA‐seq tracks include SE (*n* = 90 datasets) or PE (*n* = 40 datasets) that are formatted in six different track types. StringTie tracks show transcript models inferred by assembling RNA‐seq reads, providing the advantages of reduced data redundancy and ease of visualizing the possibility of new isoforms. Whereas predicted transcripts assembled from short RNA‐seq reads are considered to be less reliable than full‐length Iso‐Seq transcripts, the higher RNA‐seq sequencing depth leads to the StringTie tracks having the advantage of higher overall gene representation. Similar to gene predictions and Iso‐Seq, StringTie tracks allow the visualization of predicted introns and exons when zoomed in, and when zoomed out visualizations appear as histograms depicting transcript density.

Tracks based on unassembled RNA‐seq reads provide the best evidence to check the correctness of splice junctions and to confirm the need to merge gene predictions. With sufficient depth, RNA‐seq data can help determine which is correct when Ensembl and RefSeq models have a split/merge disagreement (e.g. RefSeq has one gene where Ensembl has multiple genes and vice versa). RNA‐seq junction tracks are available as arcs or as flat tracks, and can quickly reveal whether an RNA‐seq dataset would be useful in resolving a split/merge issue. The arc version is a visualization that collapses junctions from individual reads for easiest viewing when zoomed out to see an entire gene or the region between genes (Fig. 1). The thickness of the arc is related to the number of reads that support the splice junction. The flat version of the junctions track also collapses the reads, and the number of reads that support the junction is shown as the ‘Score’ when viewing details about the feature. After viewing a junctions track to determine if an RNA‐seq dataset will be helpful, the next step is to view a corresponding BAM track.

BAM tracks show alignments of individual RNA‐seq reads, and are available in two forms, dense and draggable, both of which are useful in predicting intron/exon boundaries and require sufficient zoom level to avoid an error message. The dense track (Fig. 1) is the less computationally demanding of the two BAM track types, and enables the viewing of a larger region than does the draggable track. An advantage of a draggable BAM track is that, after clicking an exon in a gene prediction track, red lines will appear at matching boundaries among all the read alignments in the BAM track. As the name indicates, draggable tracks can be dragged to the Editing Area for initiating or editing a user annotation. Right‐clicking the tracks provides more information, including details about read sequence and alignment quality.

A challenge with the availability of RNA‐seq tracks from 130 experiments is selecting the experiment with the best information for the gene of interest. A solution is to query BovineMine (Elsik *et al*. 2016; Hagen *et al*. 2018) using a transcript id (RefSeq or Ensembl) as input to retrieve expression levels in these 130 experiments, and selecting tracks from the experiments with the highest levels. An example of how to use a simple template query in BovineMine to find tissues of interest is provided in Appendix S1.

#### Tracks highlighting problematic genes and regions

Tracks provided to aid in the identification of problematic gene predictions are useful when the objective is to improve the gene set regardless of the gene family or function. The track category ‘Gene Prediction Problems’ highlights either Ensembl genes that appear to be split or merged compared with RefSeq genes or RefSeq genes that appear to be split or merged compared with Ensembl genes (Fig. 1). The track category ‘Multi‐Path Transcript Alignment’ highlights Iso‐Seq gmap alignments that may represent either chimeric Iso‐Seq sequences or genome assembly issues.

#### Private user tracks

In addition to the tracks provided by the BGD, you may view your own tracks by clicking ‘Open Track File or URL’ under the FILE menu in jbrowse. Rather than being uploaded to the BGD jbrowse/apollo server, your file remains local and is visible only in your browser alongside BGD JBrowse/Apollo tracks.

### Using blast with jbrowse and apollo

The BGD blast (Altschul *et al*. 1990) server, a custom web interface based on Sequenceserver (Priyam *et al*. 2019), is available by clicking ‘blast’ in the BGD navigation bar. It allows blast searches to be conducted against the genome using blastn (for nucleotide queries) or tblastn (for protein queries) and then viewing alignments of blast high‐scoring segment pairs (HSPs) in jbrowse. If logged into apollo, individual blast HSPs can be dragged to the Editing Area to start a new annotation. The blast server is especially useful for identifying the locations of bovine genes based on their homologs, for example, from model organisms such as human or mouse.

### General process of annotating a gene at the BGD

Here we describe in general terms how to annotate a gene using BGD tools in order to convey what the annotation process entails. Detailed step‐by‐step examples are provided in Appendices S1 and S2.

Prior to starting annotation, identify a gene or genomic region of interest, for example, by searching with a RefSeq or Ensembl transcript id, or by performing a blast search with a bovine cDNA or protein homolog sequence. After navigating to the genomic region containing the gene of interest, select tracks to display the gene model and isoforms using the Faceted Track Selector. First inspect the RefSeq and Ensembl tracks for differences across the gene sets. If there is no major disagreement, such as when one gene set indicates two genes and the other indicates one (a split/merge issue), the annotation can be initiated using the RefSeq and Ensembl transcripts. The goal is to annotate an entire gene, if possible, which entails annotating all of the transcript isoforms for which there is evidence. To begin, select a unique set of isoforms from the RefSeq and Ensembl tracks, but do not select isoforms with major incongruences compared with others without further inspection. Drag each selected isoform to the Editing Area after right‐clicking an intron. Sometimes two isoforms are identical across genes sets, in which case select only one. Sometimes an isoform in one gene set will appear to be a fragment of an isoform found in the other, in which case, select the longer isoform. There is no need to be overly concerned about the choice of isoforms at this point, because each isoform can be easily deleted from the Editing Area.

The next step is to view transcriptome data to look for new isoforms. First determine whether the gene is present in the Iso‐Seq Combined PASA track and whether the track reveals new isoforms. If the gene is absent from the Iso‐Seq track, skip to the next step, visualization of RNA‐seq. If the gene is present in the Iso‐Seq track, use the two‐letter codes in the transcript labels to identify tissues and then investigate the corresponding Iso‐Seq gmap tracks for each tissue (Table S2). If an Iso‐Seq alignment appears to be unusual, for example, with an unusually long internal exon, RNA‐seq tracks should be viewed (the next step) to confirm its expression. Whether or not the gene is present in the Iso‐Seq data, use BovineMine to identify RNA‐seq tracks with the highest expression levels for the gene of interest. Once suitable tissues have been identified, the corresponding StringTie tracks will provide a quick way to identify potential new isoforms. The dense version of the corresponding RNA‐seq BAM track should then be investigated to further support the presence of alternative exons, as StringTie may produce spurious transcripts with a low depth of read support. Any new isoforms identified with Iso‐Seq and StringTie should be added to the Editing Area.

Once isoforms have been added to the Editing Area, you can make several types of changes, including adding or extending UTRs, checking splice sites or translation start/stop sites and editing exons. Right‐clicking the intron of an annotation opens a menu with several editing options, as well as ‘Get Sequence’ to retrieve the protein, coding sequence (CDS) or cDNA sequence; ‘Show History’ to see modifications made; ‘Undo/Redo’ to undo or redo the previous edits; and ‘Edit Information’ to add more information about the annotation. apollo provides visual cues to help with exon editing. Red highlights appear at exon boundaries in other tracks when they agree with the exon boundaries of the selected isoform (Fig. 2). Modifications that change the reading frame cause coding exons to change color. If a modification introduces an early stop codon, some of the coding exons will change in appearance to be represented as UTRs.

After edits to the annotation are complete, the next step is to obtain the sequence and search a well‐curated protein database such as UniProt/SwissProt (UniProt Consortium 2019), to confirm that the annotation is congruent with known protein sequences. Alignments to known proteins can reveal truncated, merged or incorrectly extended annotations. Performing a BLASTX search with a CDS enables the detection of reading frame shifts. In the best case scenario, the CDS to protein alignment would cover the entire lengths of both the CDS and the protein sequence in a single alignment. A CDS alignment to part of a protein suggests that the annotation is truncated, but sometimes there is insufficient evidence to further improve the annotation. An incorrectly merged annotation is indicated when part of the CDS aligns to one protein and another part aligns to an unrelated protein. Any part of the CDS alignment that occurs in a reading frame other than plus one indicates a frame shift in the annotation. Further editing of the annotation is required when a protein comparison suggests an incorrect merge or frame shift. Searching for protein domains in the interpro database can provide additional evidence of protein sequence integrity (Mitchell *et al*. 2019).

After completing the annotation process, you may have determined that the original predicted isoforms needed no modification and that there were no new isoforms; nevertheless, the gene annotation is important because it indicates that a predicted gene has been reviewed and confirmed. Whether or not you modified the annotation, you can provide additional information about the gene using the Information Editor, available by right‐clicking the annotation. The form provides text entry boxes for gene symbol, description, database cross‐references, GO, PubMed identifiers and comments. For gene symbols, you should use standard nomenclature established by the Vertebrate Gene Nomenclature Committee (Braschi *et al*. 2019) whenever possible. Use the comments section to indicate if the finished annotation appeared to be truncated or had other unresolvable issues, or if only a subset of isoforms were annotated.

Throughout the annotation process, the server automatically saves annotation changes and the history of changes at each step. Both the annotation and change histories are available to all logged in users. Any user can make edits to a previous annotation or delete the annotation from the editing area, but you should never delete another’s work as the history will also be deleted. The annotation owner, visible by hovering over the annotation, should be contacted if changes need to be made to the annotation.

## Conclusion

With the release of a new highly contiguous bovine reference genome assembly, a collaborative effort to manually annotate genes would greatly enhance the bovine community’s genomics resources. Gene models curated using the BGD apollo tool will be immediately available to the bovine research community, and will periodically be submitted to NCBI for inclusion in future bovine genome annotation updates. Whether the objective is overall gene set improvement or examining specific genes of interest, we encourage the research community to use the freely accessible annotation tools at the BGD and we welcome suggestions to improve the utility of the resource.

## Supporting information


**Table S1** RNA‐seq sample metadata.

**Table S2** Two‐letter codes for Iso‐seq pasa combined assembly track.

**Appendix S1** Annotation example 1.

**Appendix S2** Annotation example 2.

## Data Availability

RNA‐seq data are available at NCBI (BioProjects PRJNA263600 and PRJNA294306). The bovine genome annotation tools described here are freely available at the Bovine Genome Database (http://bovinegenome.org).
