# Supplementary material for: Using online tools at the Bovine Genome Database to manually annotate genes in the new reference genome
Source: Anim Genet. 2020 Jun 14;51(5):675–82. doi: 10.1111/age.12962 (PMC7540445; doi:10.1111/age.12962)
Supplement: Supplementary file 2 — Table S2 Two‐letter codes for Iso‐seq pasa combined assembly track. [file AGE-51-675-s002.docx]

Table S2. Two Letter Codes for Iso-Seq PASA Combined Assembly Track

| **Tissue** | **Code** |
| --- | --- |
| Medulla oblongata | MO |
| Left lung | LU |
| Thalamus | TH |
| Subcutaneous fat | SF |
| Lymph nodes | LN |
| Liver | LI |
| Jejunum | JE |
| Hypothalamus | HY |
| Testis | TE |
| Temporal cortex | TC |
| Longissimus dorsi (ribeye/loin) | LD |
| Cerebral cortex | CC |
| Mammary gland | MG |
| Duodenum | DU |
| Atrium | AT |
| Abomasum | AB |
| Reticulum | RE |
| Omasum | OM |
| Uterine myometrium | UM |
| Ventricle | VE |
| Aorta | AO |
| Rumen | RU |
| Lactating mammary gland | LM |
