# Supplementary material for: Using online tools at the Bovine Genome Database to manually annotate genes in the new reference genome
Source: Anim Genet. 2020 Jun 14;51(5):675–82. doi: 10.1111/age.12962 (PMC7540445; doi:10.1111/age.12962)

APPENDIX S1 – ANNOTATION EXAMPLE 1

This example shows the annotation of *TBRG1*, Transforming Growth Factor Beta Regulator 1, with the addition of new transcript isoforms and the investigation of a questionable coding sequence prediction in an existing RefSeq transcript.

Non-registered users can try this example using the Bovine Apollo Demo instance available via the Apollo pulldown menu in the BGD navigation bar (Fig S1). Keep in mind that other readers may also be trying this example. If you follow the instructions below and find that the gene of interest is already annotated, you can mouse over the annotation to determine the date and time the annotation was submitted. If it has been over a day, you can delete the annotation(s) by right clicking an intron and select Delete. **This instruction to delete an existing annotation that you did not create yourself pertains only to the *Bovine Apollo Demo* instance accessed as shown in Fig. S1!**

Figure S1. Accessing Bovine Apollo Demo.

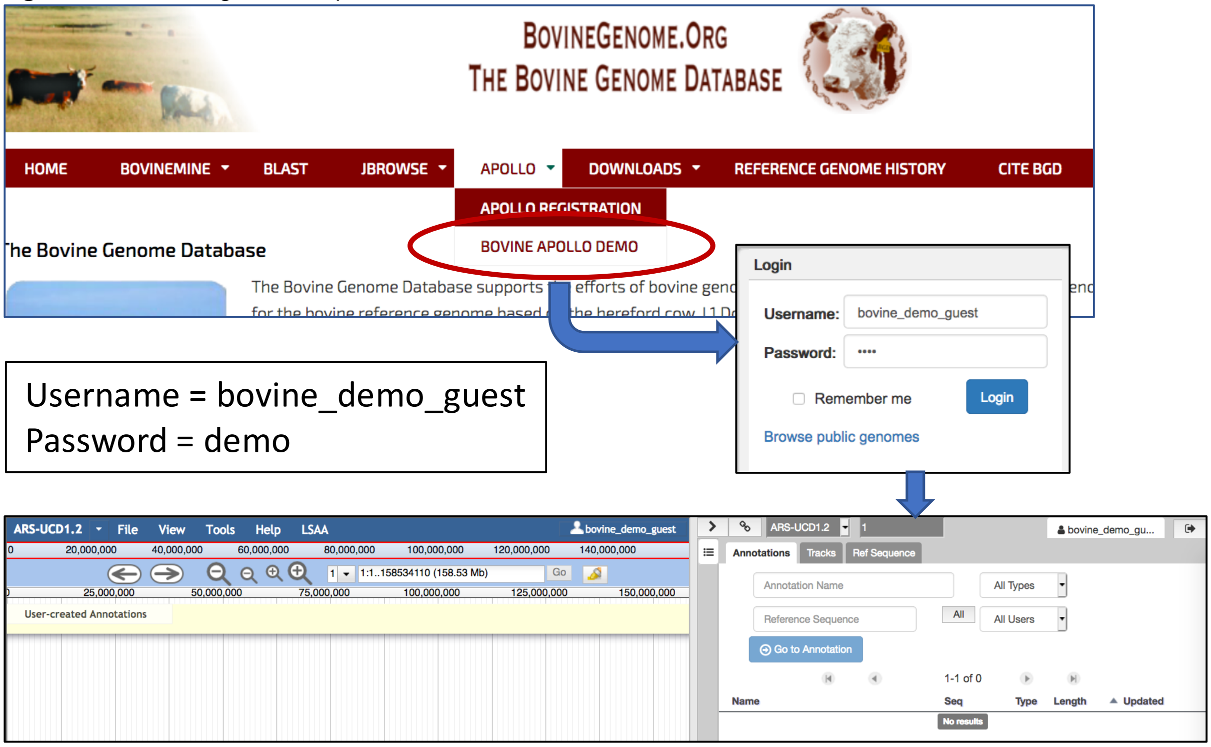

Registered users can login to the main Bovine Apollo instance by first accessing JBrowse for ARS-UCD1.2 using the JBrowse pulldown menu in the BGD navigation bar, and then logging in to Apollo using the login button in the upper right corner (Fig. S2). **Note that if you access the main Bovine Apollo instance using a registered account, you <u>cannot</u> follow along with this example, as it would require deleting user annotations!**

Figure S2. Accessing Apollo using a registered account.

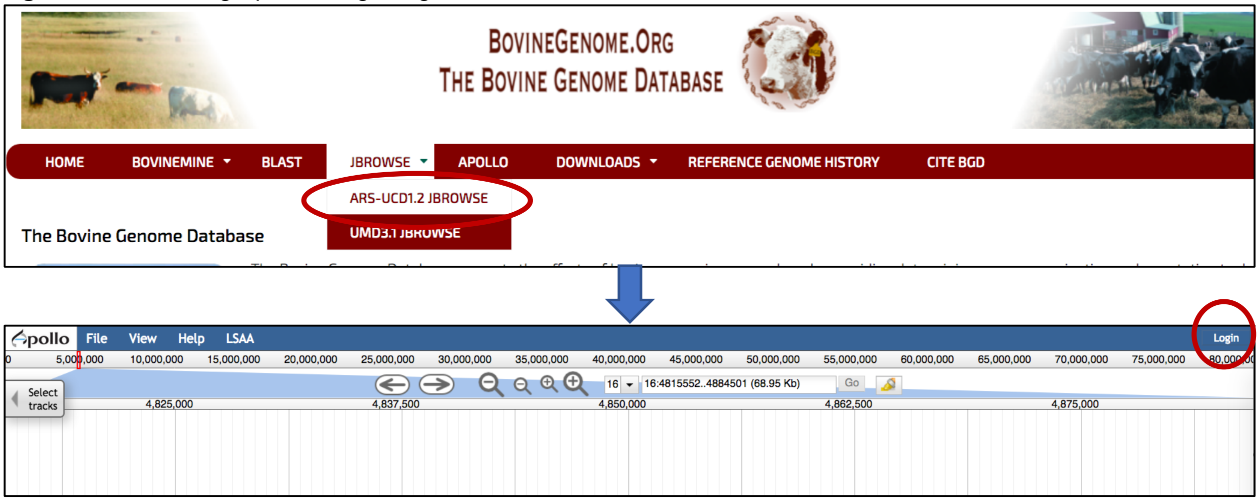

Whether you have logged in to the Bovine Apollo Demo or the main Bovine Apollo, after Apollo is loaded into your browser, you should prepare your browser view for annotating. Use the greater-than sign in the upper left of the Information Panel to hide the Information panel and increase the view of the browser. Bring the Select Tracks tab for the Faceted Track Selector into view by clicking the icon that looks like a list below the less-than sign in the upper far right of the browser (Fig S3).

Figure S3. Process of setting up the browser for annotation. Clicking the greater-than sign to the left of the Information panel hides the panel. Clicking the list icon brings the Select Tracks tab back into view for access to the Faceted Track Selector. Note there are three additional tabs in the track panel compared to what most users will see, because the extra tabs for Users, Group and Admin are available only to BGD Apollo administrators.

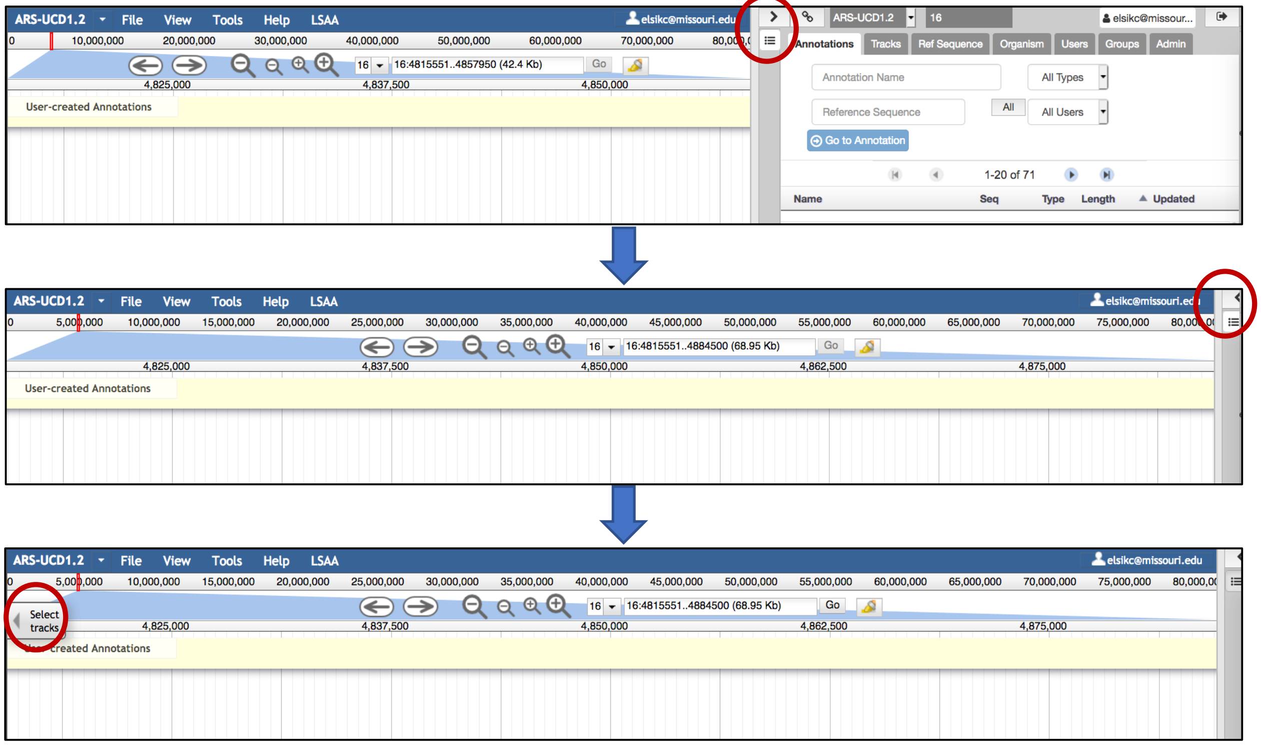

Prior to navigating to a gene of interest in Apollo, retrieve a bovine transcript id for *TBRG1* using BovineMine. In the navigation bar on the BGD home page, use the BovineMine tab to access the latest version of BovineMine (currently BovineMine v1.6). The Template Tabs in the middle of the BovineMine home page show query categories (Fig. S4).

Figure S4. BovineMine home page. The template query bar in the middle of the page provides tabs with template queries organized into categories.

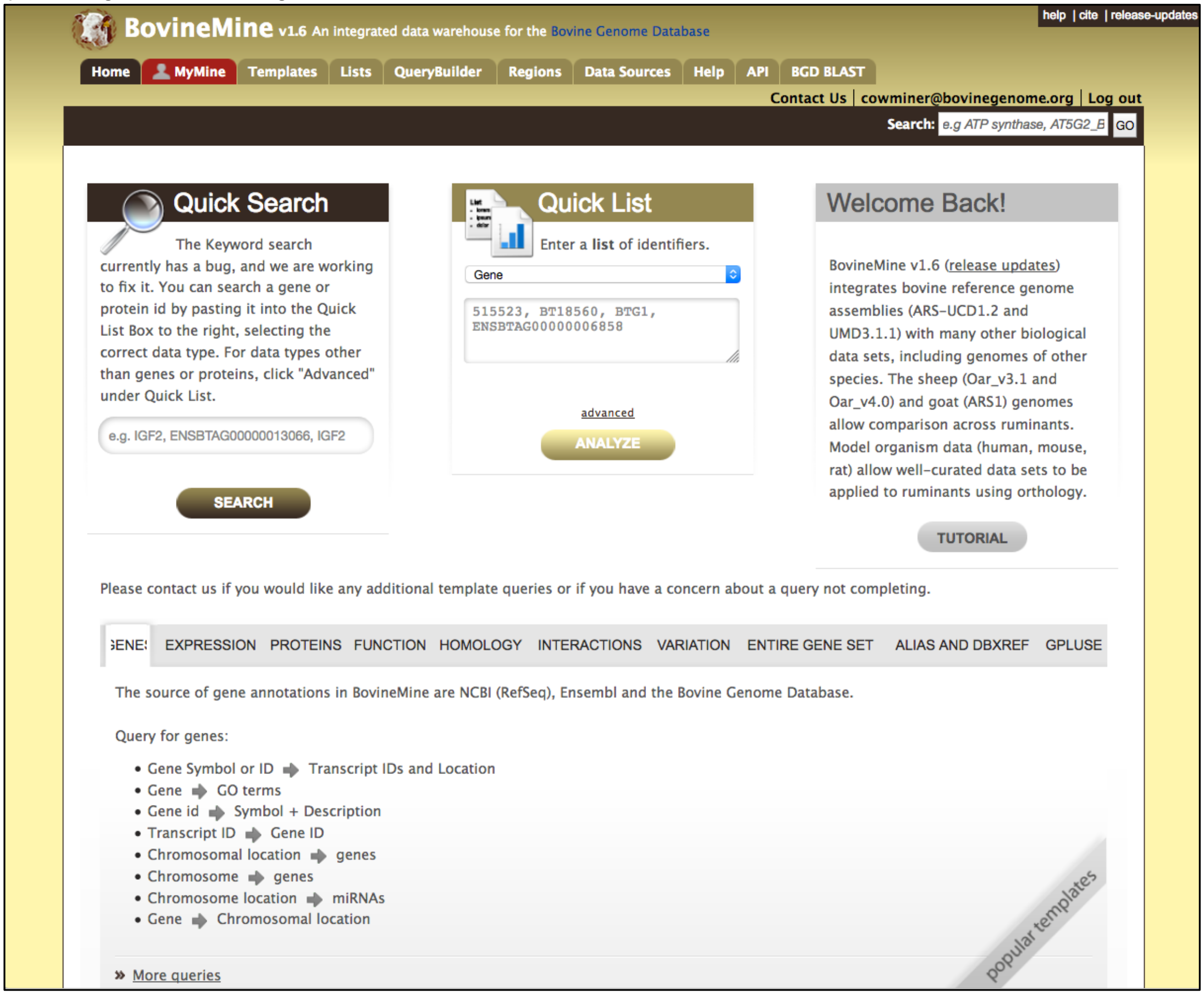

In the Genes category, look for the template query called "Gene Symbol or ID → Transcript IDs and Location" (Fig. S5). If it does not appear in the list showing on the home page, click "more queries" to see the entire list of gene related queries. Once the correct query is located, click its name to open the template query form. Enter "TBRG1" into the box next to "Lookup" and make sure "B. taurus" is selected as the organism. For the gene *source*, select either RefSeq or the Ensembl release provided. We selected RefSeq and found NM_001075315.1 as one of the identifiers. Note that for RefSeq, ids that begin with "NM_" or "XM_" are used for protein coding transcripts, and those with "NR_" or "XR_" are used for non-coding RNA.

Figure S5. Process of using a template query to retrieve a transcript id for a given gene symbol.

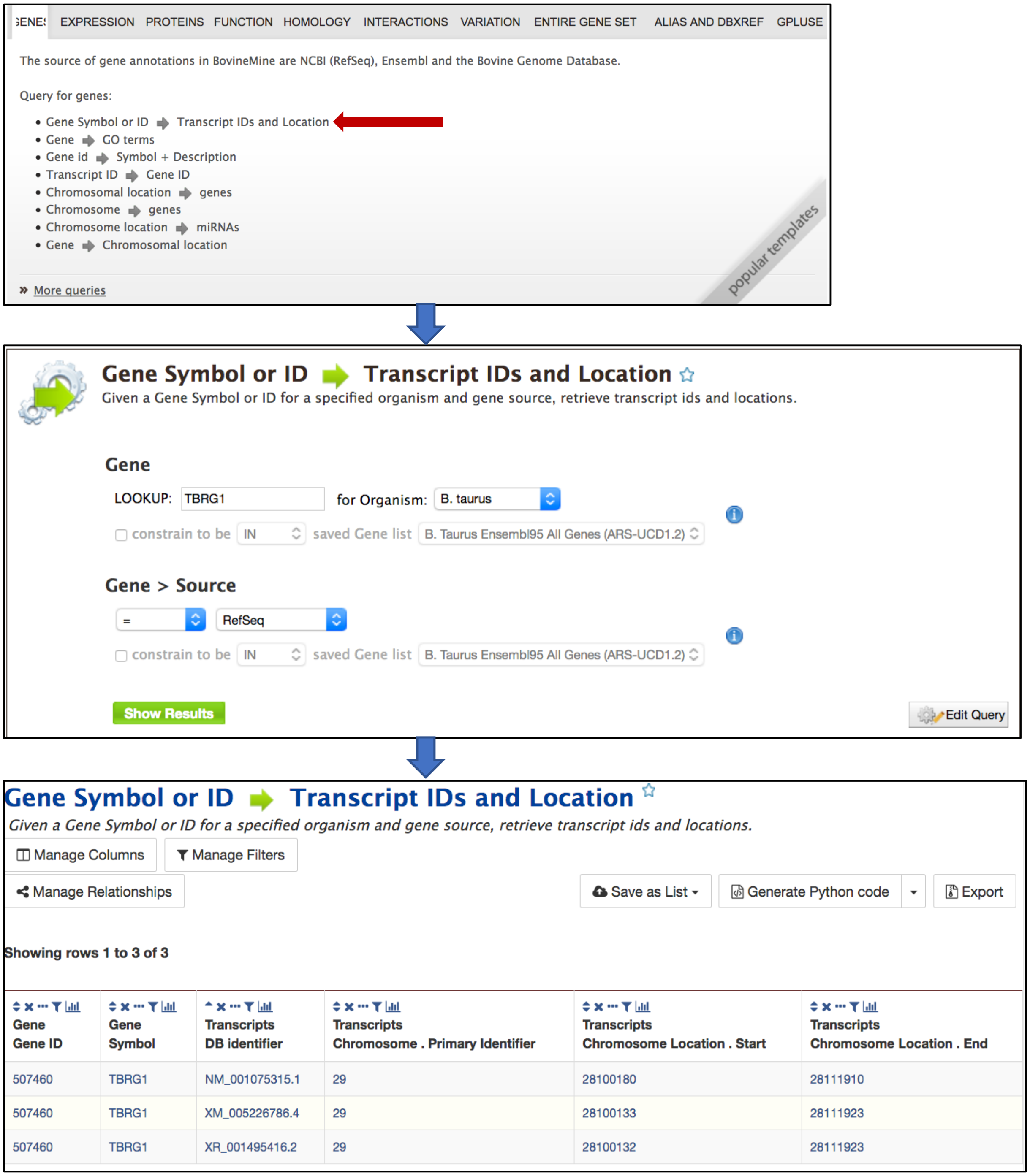

Once a transcript id is identified, go back to Apollo. Enter the RefSeq transcript ID (NM_001075315.1) into the navigation search box then click “Go”. Two tracks show up with the same feature id both in the same location on chromosome 29. One track is the current RefSeq protein coding gene set and the other track shows genes from the previous genome assembly that have been mapped onto ARS-UCD1.2. Click the “Show” button next to RefSeq Protein Coding to go to the location and display the *RefSeq Protein Coding* track in JBrowse (Fig. S6).

The glyphs representing the transcripts in the browser show exons as rectangles with introns as lines connecting the rectangles. Arrows at the 5’ ends of the transcripts indicate the direction of transcription. Transcripts pointing from the left to right are transcribed on the plus strand, and those pointing from right to left are transcribed on the minus strand. Introns in mammalian transcripts are so long compared to exons that the exons often appear as small ticks when viewing the entire length of the transcript. Zooming in further reveals the rectangular shape and different colors of the exons. For protein-coding transcripts at BGD, untranslated regions of exons are shown in dark blue, while coding exons are slightly thicker rectangles in three different colors representing different reading frames (Fig. S6).

Figure S6. Searching with a transcript id and the result after clicking “Show”.

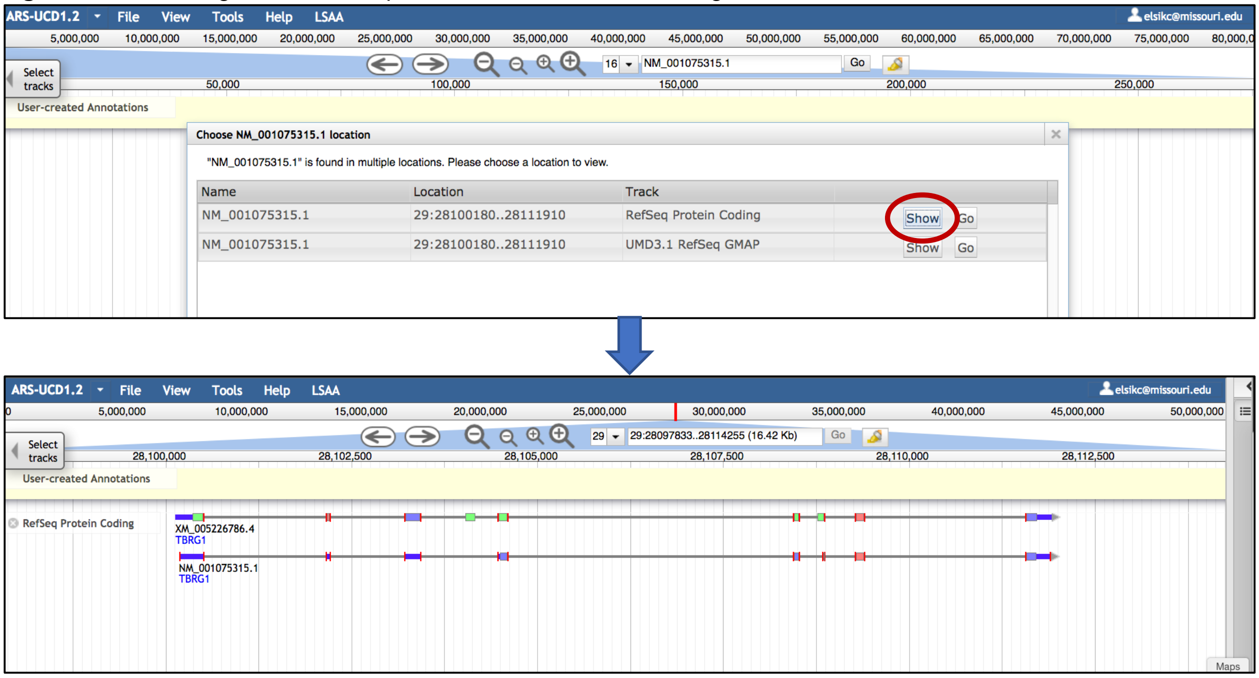

Click the “Select tracks” tab to access the Faceted Track Selector. The Table of tracks shows that the RefSeq Protein Coding track is already selected. Click the *Gene Prediction* and the *Iso-Seq Combined PASA Assembly* categories to narrow the list shown in the table on the right. Add two more tracks to the browser by checking the boxes next to Ensembl Protein Coding and Iso-Seq Combined PASA Assembly. Click “Back to browser” to see both RefSeq and Ensembl Protein Coding genes in the browser view (Fig. S7).

Figure S7. Selecting tracks using the Faceted Track Selector and the resulting tracks shown in the browser.

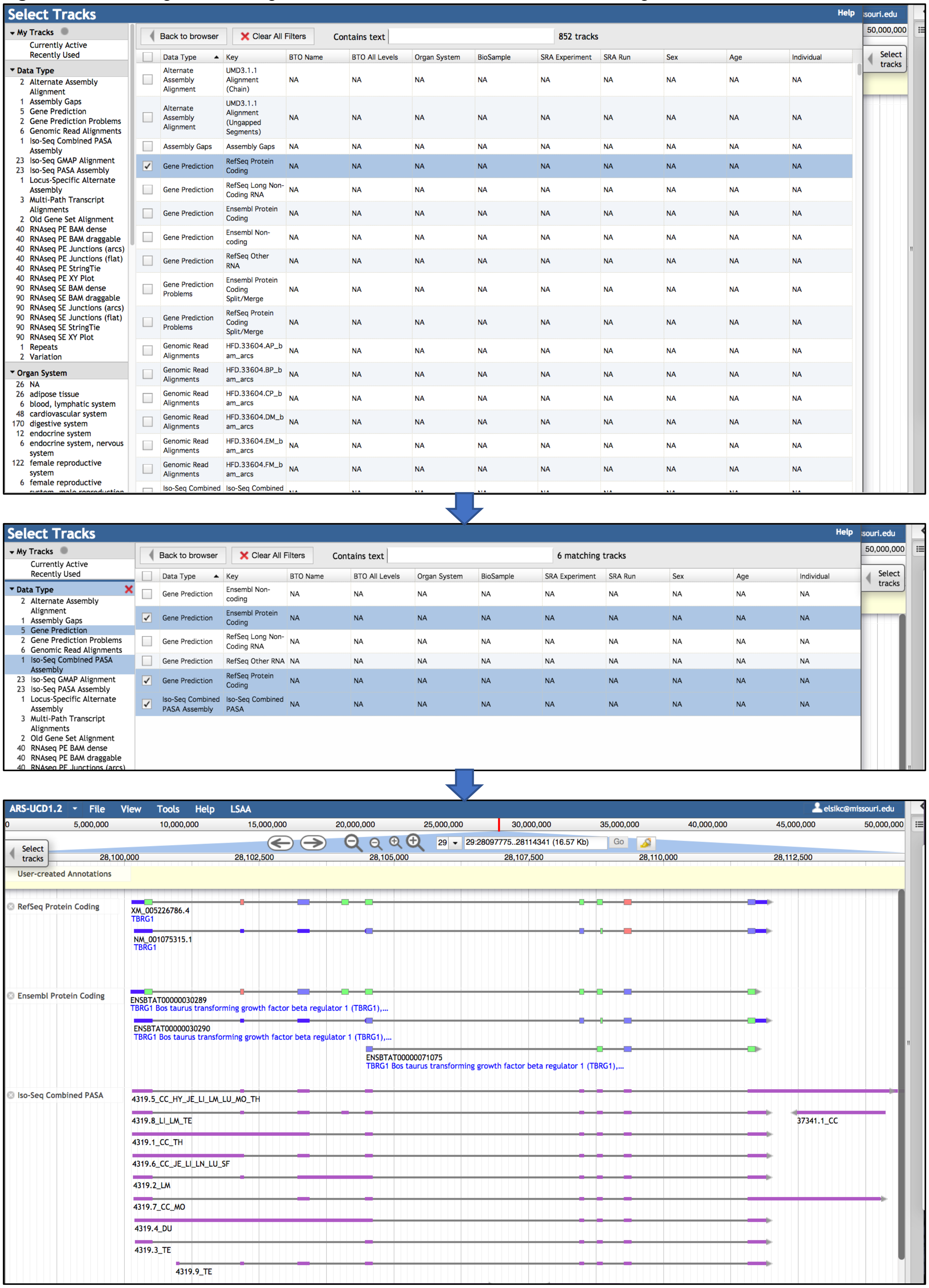

Inspection of the *RefSeq Protein Coding* and *Ensembl Protein Coding* tracks suggests that the longer predicted transcripts are similar across gene sets. Clicking an intron of the RefSeq transcript XM_005226786.4 allows both dragging the transcript to the Editing Area and highlighting the boundaries of the exons. Matching red lines at all the exon boundaries in the exons of Ensembl transcript ENSBTAT00000030289 show that the intron/exon structure is the same as that in XM_005226786.4 (Fig. S8). A similar comparison between RefSeq NM_001075315.1 and Ensembl ENSBTAT00000030290 shows they are also the same. Therefore, drag only one set to the Editing Area. In this example, we used the RefSeq set. At this point, we will ignore the short Ensembl transcript (ENSBTAT00000071075), because it appears to be a fragment, but it may be worthwhile to investigate further at some point.

Figure S8. Apollo view after dragging a transcript to the Editing Area. After clicking the transcript, it is outlined in red, and red edges are shown on all exons in other tracks with splice sites that match those in the outlined transcript.

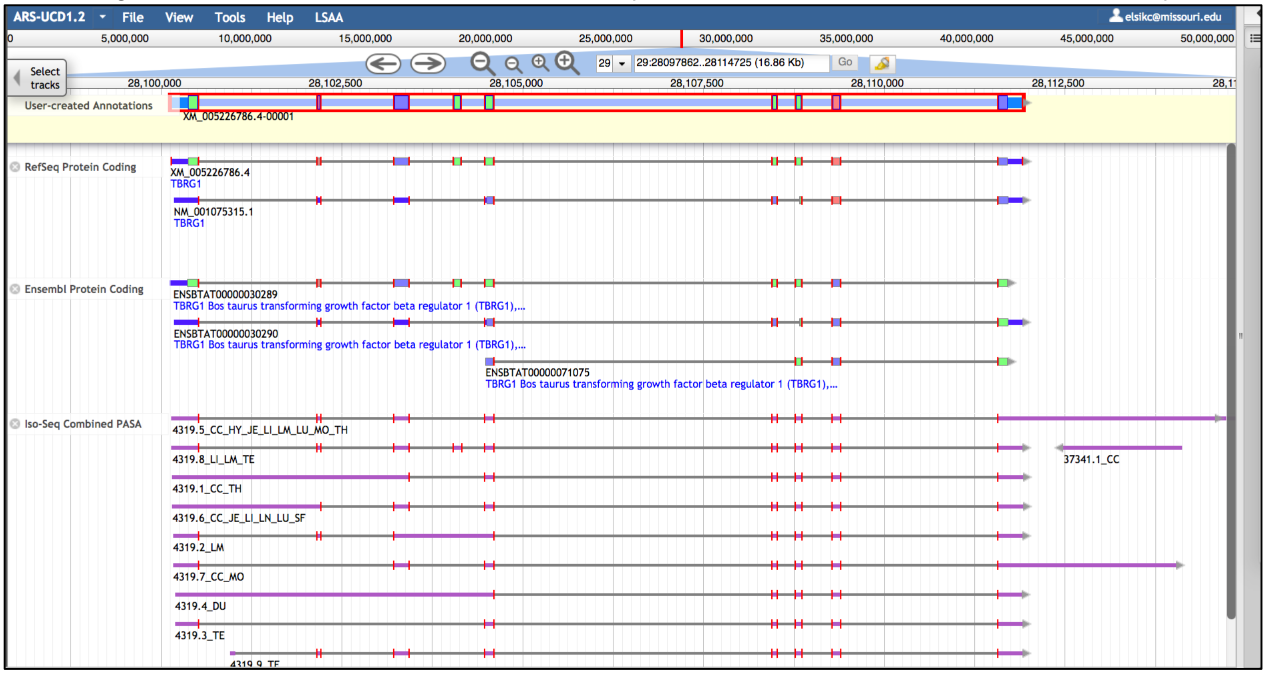

After dragging the predicted transcripts to the editing area, remove the *RefSeq Protein Coding* and *Ensembl Protein Coding* tracks from the viewing area by clicking the X in the track labels. The next step is to inspect the *Iso-Seq Combined PASA* track. To make it easier to detect differences between the Iso-Seq transcripts and the predicted transcripts, drag all of the Iso-Seq transcripts to the Edit Area (Fig. S9). Notice that the temporary unique identifiers of all transcripts are based on the id of the first transcript added to the Editing Area (XM_005226786.4), with incrementing numerical extensions added to each transcript. There is no need to be concerned about these temporary identifiers, as they will be reassigned when manual annotations are incorporated into the gene set at BGD.

Determine whether any of the Iso-Seq transcripts have identical exon structure by clicking an intron to highlight each predicted transcript and compare it to all the isoforms. Because XM_005226786.4-00004 is identical to XM_005226786.4-00001, either of these can be deleted by right clicking an intron, then selecting Delete in the pulldown menu (Fig. S10). In this example, we deleted XM_005226786.4-00004.

Figure S9. Apollo view after all transcripts have been dragged to the Editing Area. Each transcript is clicked to highlight exon edges that match with other transcripts. This shows that XM_005226786.4-00001 is identical to XM_005226786.4-00004, so the latter can be deleted.

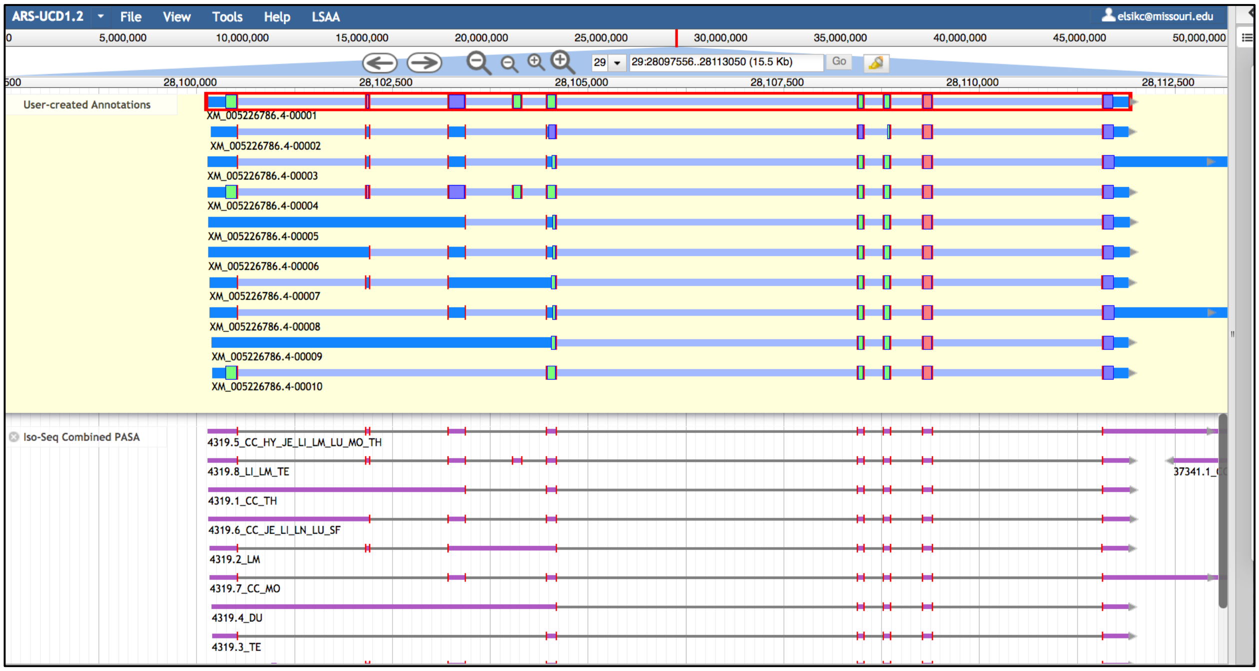

Figure S10. Deleting transcript XM_005226786.4-00004.

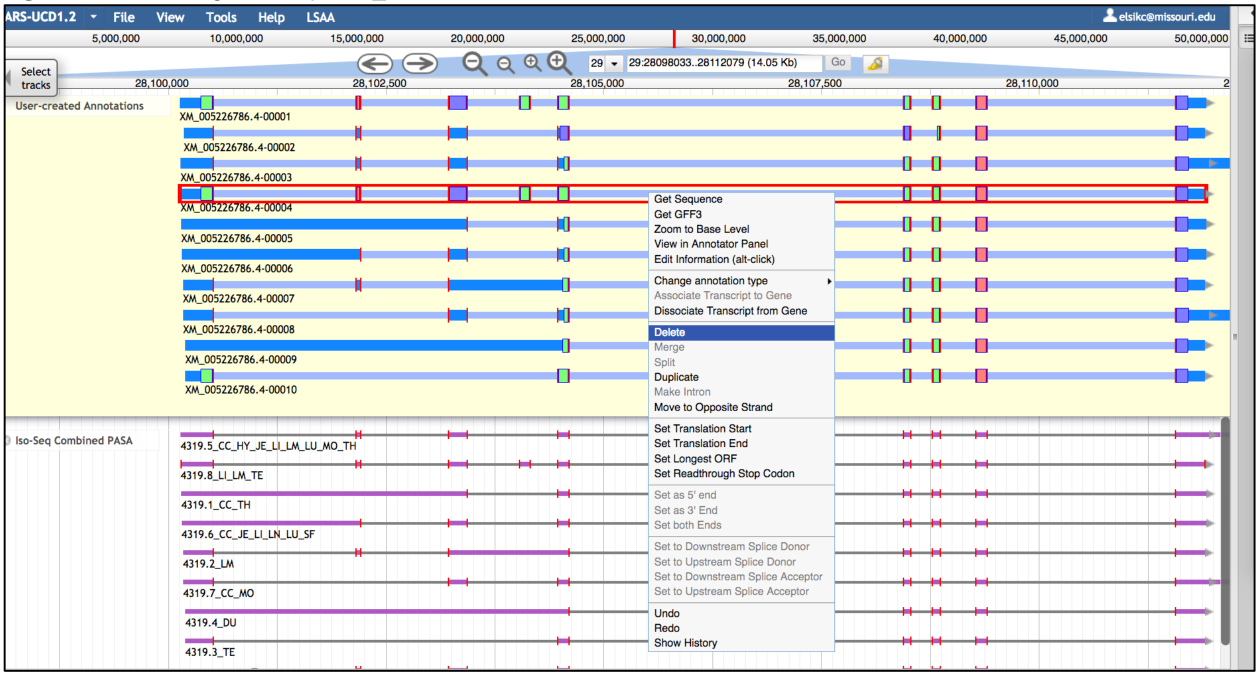

Further inspection of the coding exons in the Editing Area shows that the reading frame of exons 4 and 5 in XM_005226786.4-00002 disagree with those of all the other transcripts, as indicated by different coloration of the exons. In addition, the start codon in exon 4 of XM_005226786.4-00002 appears to be upstream of the start codons in isoforms -00003, -00005, -00006, -00007, -00008 and -00009, as indicated by the location of the boundary between the untranslated regions (UTR) in blue and the coding portion of the exons in purple or green. When reading frames disagree among isoforms, BLASTX comparison to well-known proteins can reveal which is correct. In this example, we start with the first isoform, XM_005226786.4-00001. Right click an intron then select Get Sequence in the pulldown menu. In the resulting Sequence box, select CDS sequence (Fig. S11).

Figure S11. The process of obtaining the CDS sequence for XM_005226786.4-00001.

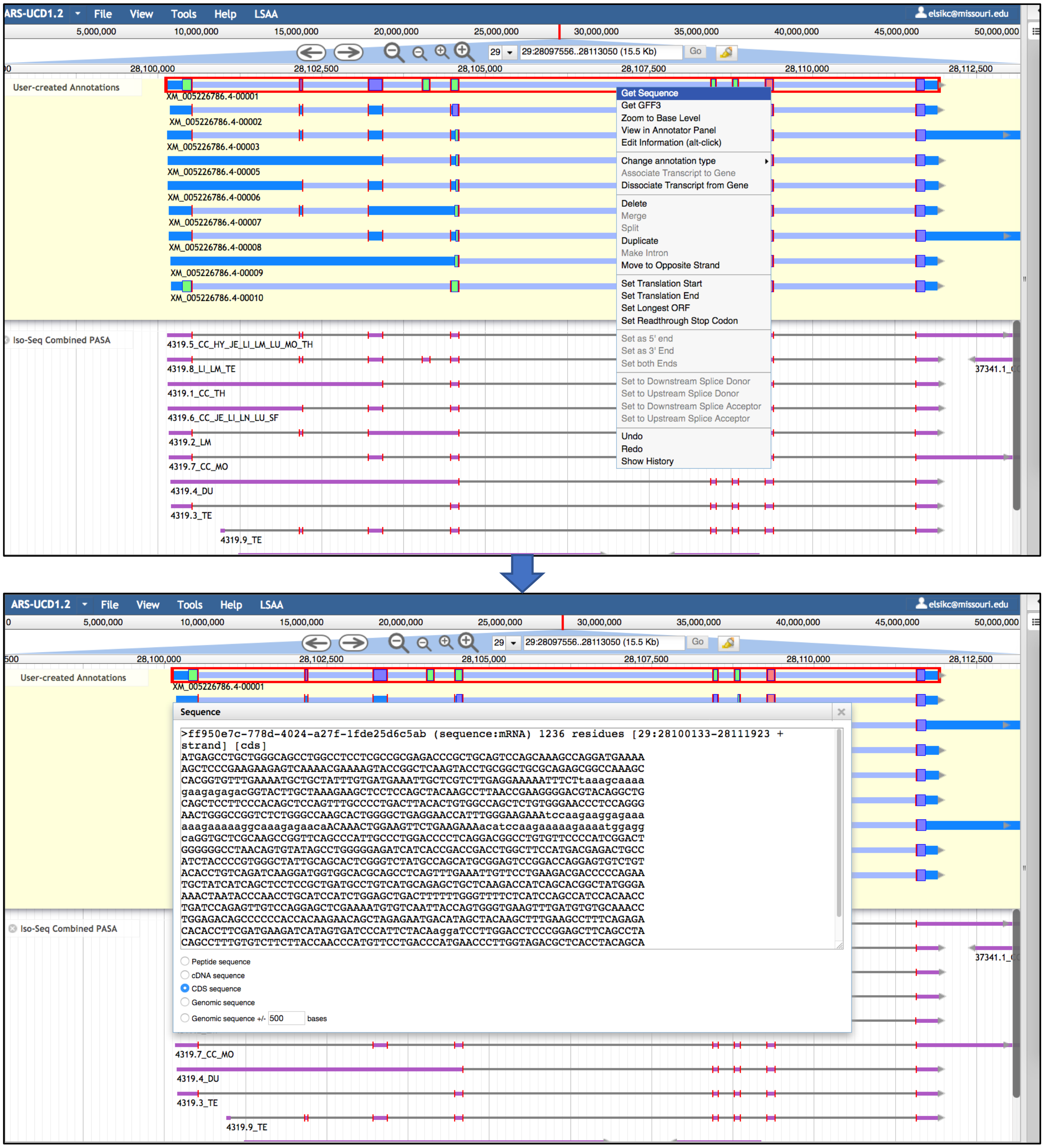

Go to the NCBI BLAST web page (https://blast.ncbi.nlm.nih.gov/Blast.cgi) and select BLASTX. Paste the CDS sequence in the form and select "UniProtKB/Swiss-Prot(swissprot)" in the Database pulldown menu. Click "BLAST" to begin the search and wait for the results (Fig. S12). In the results page, just above the list of hits, select the Alignments tab. The first alignment shown is to a homologous human protein with a length of 411 amino acids. The beginning of the alignment is at position 1 in both sequences, and the end of the alignment is at position 1197 in the CDS and 399 in the human protein, thus nearly full-length of both sequences. Also note that alignment occurs in a single segment in reading frame +1, which is expected if the predicted CDS is correct (Fig. S13).

Figure S12. BLASTX input page with UniProt/Swiss-Prot database selected.

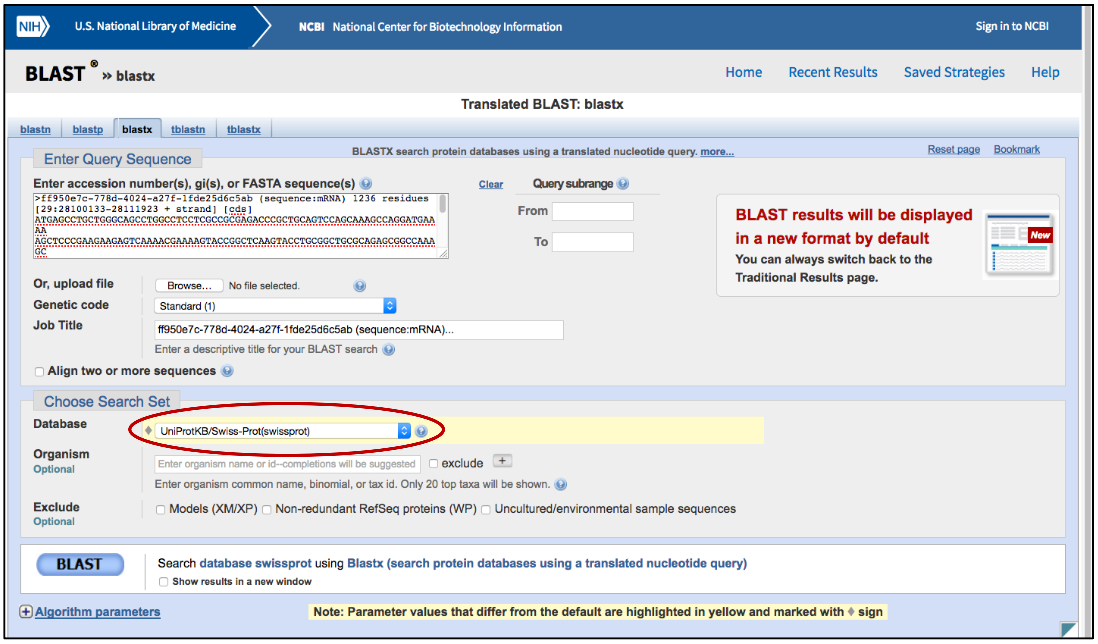

Figure S13. BLASTX alignment of the predicted CDS to a known human protein showing nearly full-length match and +1 reading frame.

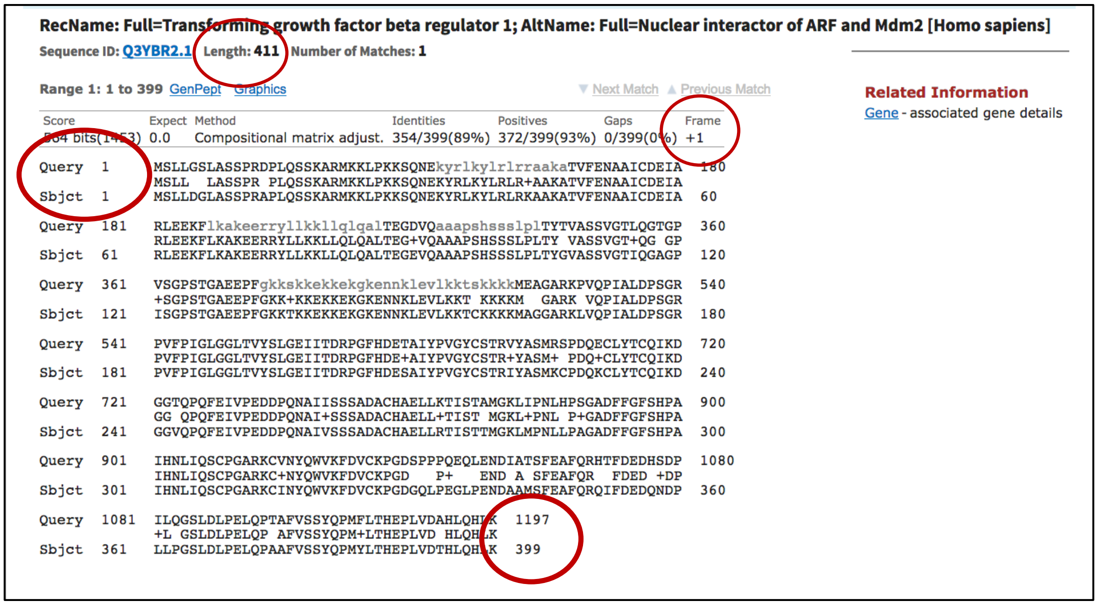

The BLASTX search is repeated using the CDS sequence of XM_005226786.4-00002 (Fig. S14). The resulting alignment shows two match segments. The match with the higher score is shown first, and corresponds to the 3' end of the CDS sequence. Notice that the second match is in reading frame +3, meaning that introduction of a deletion or insertion is needed in order for the CDS to translate into a protein that has structure to the human protein (Fig. S15). Without a further sequence change, the CDS would code for an amino acid sequence at positions 3 to 221 that is not homologous to the human protein sequence. Since it is highly unlikely that a protein with such a drastic change in amino acid content compared to the known protein could fold into a functional secondary or tertiary structure, it is assumed that this CDS prediction is incorrect.

Figure S14. View of transcripts in Editing Area, highlighting XM_005226786.4-00002 which has coding exons with reading frames that disagree with all other transcripts, as shown in different colors.

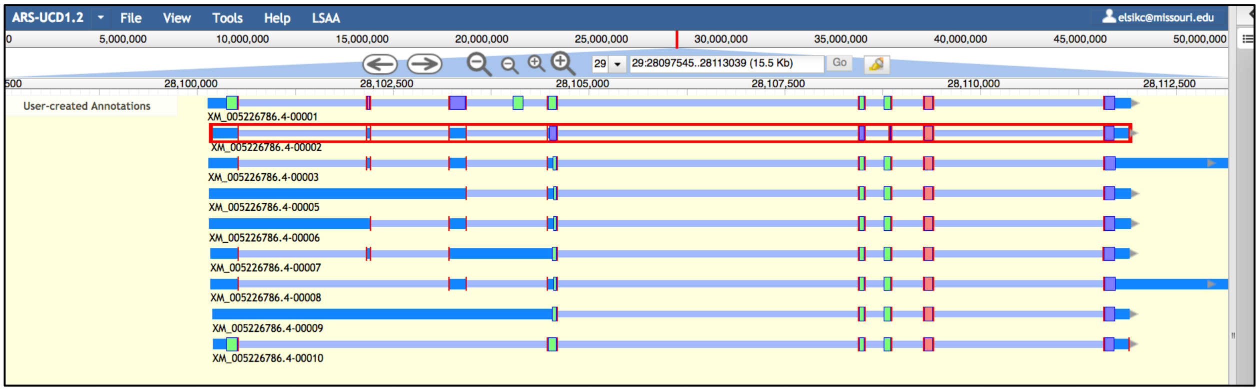

Figure S15. BLASTX alignment of XM_005226786.4-00002 to known human protein showing only partial alignment and reading frame shift.

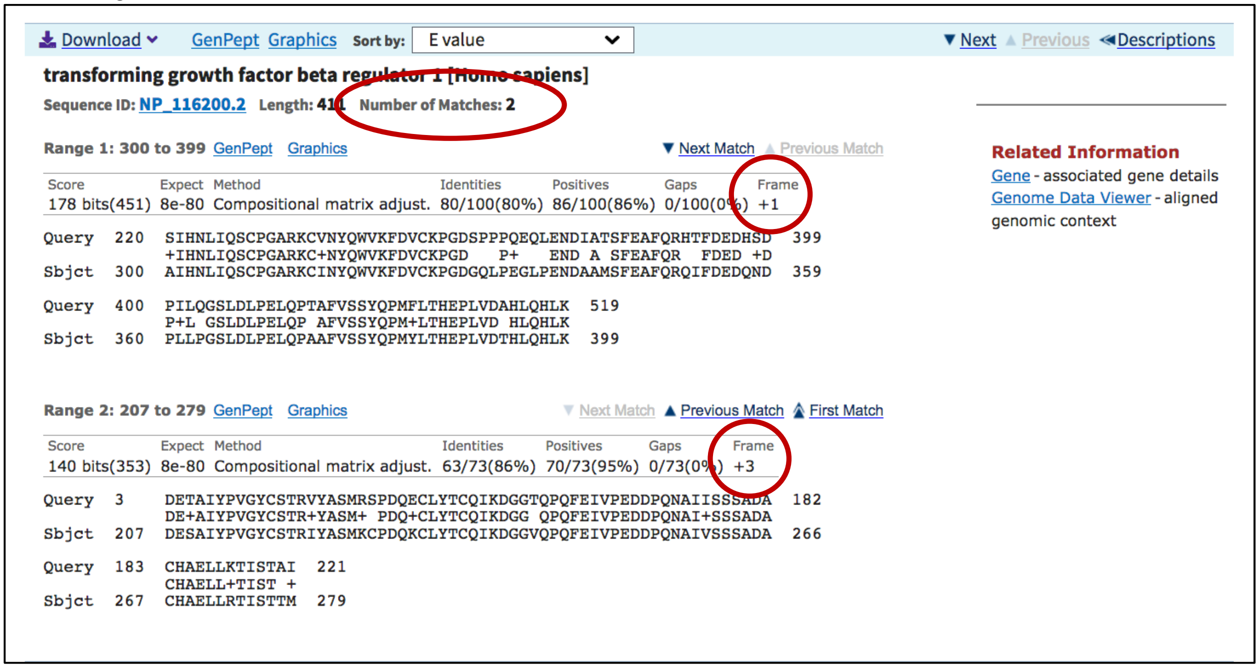

Back in Apollo, determine whether a change in the translation start site of XM_005226786.4-00002 will correct the exon reading frames so that they agree with the known protein. To do so, center the browser onto an exon and zoom in using the large + button in the navigation area until sequence appears. Double stranded genomic DNA sequence is shown in between colored lines of amino acid sequences. The upper

DNA strand is the plus strand (shown left to right in 5' to 3' direction) and the lower line is the complementary minus strand (shown left to right in 3' to 5' direction). The three colored amino acid lines above the DNA sequence represent the three plus strand reading frames, and the three colored amino acid lines below the DNA sequence represent the three minus reading frames. The coding exons in the Editing Area are colored coded to match the reading frame. For example, to determine the amino acid sequence coded by the green exon in Fig. S16, look at the amino acid sequence in the green line above the DNA sequence. Within the amino acid lines, methionines (M), which are coded by start codons (ATG), are highlighted in yellow. Stop codons are shown as asterisks (Fig S16).

To investigate each exon in this example, look for start codons in the top three (plus strand) reading frames, note whether there is a nearby downstream stop codon, and note whether the background color (representing reading frame) is the same as the exon color of XM_005226786.4-00001, which matched well to a known protein. Investigating each exon in this way shows three possible start codons in exon 1 (Fig. S16). The most upstream start codon is in the "purple" reading frame and has a nearby stop codon. The next start codon is in the same position and reading frame as the start codon in transcript XM_005226786.4-00001, making it a good candidate. The codon in the most 3' prime position of exon 1 is also in the same reading frame as XM_005226786.4-00001, but the previous start codon is a better candidate because it would code for a more complete protein. The same type of investigation is performed for the remaining exons. In addition to the three start codons in exon 1, we found the following: one start codon in exon 2 (incorrect frame), four start codons in exon 4 (one with correct frame that is equivalent to the start codons in transcripts -00003, -00005, -00006, -00007, -00008 and -00009), three codons in exon 5 (all incorrect frame), three codons in exon 7 (all incorrect frame), one start codon in exon 8 (correct frame, but close to the stop codon).

Figure S16. Zoomed in view of first exon, showing potential start codons, two of which are in a reading frame (green) that agree with other transcripts.

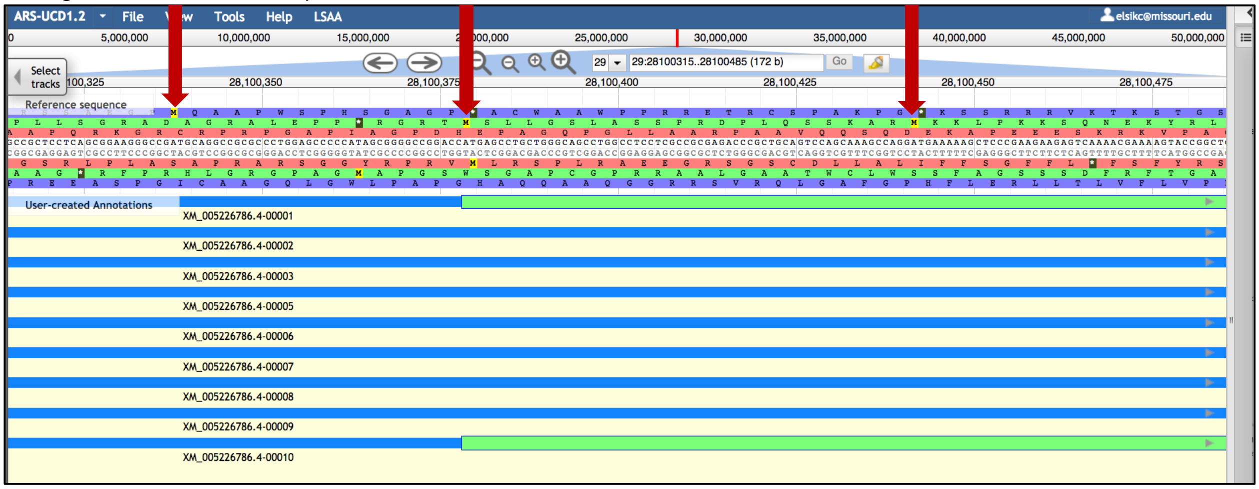

The next step is to reset the translation start. We choose the second start codon in exon 1 because it matches the start codon in XM_005226786.4-00001. Once zoomed, reset the start codon of XM_005226786.4-00002 by clicking the exon so that DNA sequence appears within the exon. Then right click the selected ATG, and select Set Translation Start in the pulldown menu (Fig. S17). After resetting the translation start, the coding part of the exon turns green, and agrees with XM_005226786.4-00001 and XM_005226786.4-000010 (Fig. S17). Zooming out shows that the color of the first three coding exons have changed and agree with XM_005226786.4-00001, but a small part of exon 4 is still disagrees with XM_005226786.4-00001 and a stop codon is introduced so that the 3' untranslated region (UTR) now starts in exon 4 (Fig. S17).

Figure S17. Process of resetting the translation start to first exon and Apollo view showing result.

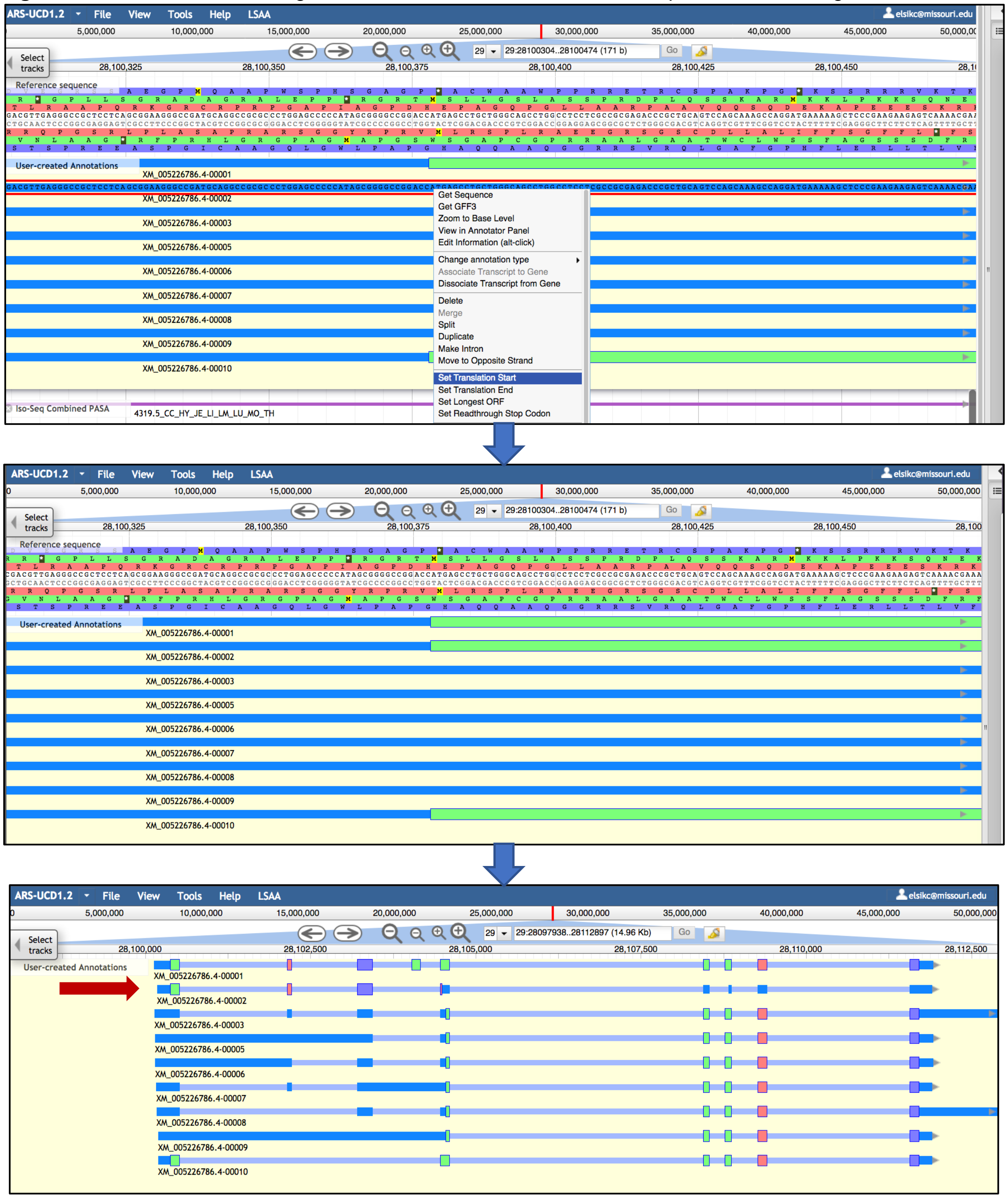

Performing a BLASTX search with the modified CDS of XM_005226786.4-00002 shows a good alignment to the N-terminal region of the human homolog (Fig. S18). Although the predicted protein appears to be a fragment compared to the known protein, the modified protein isoform is more plausible than the original isoform because its translation agrees with a known protein.

Figure S18. BLASTX alignment of modified XM_005226786.4-00002 to known human protein with no indication of a reading frame shift.

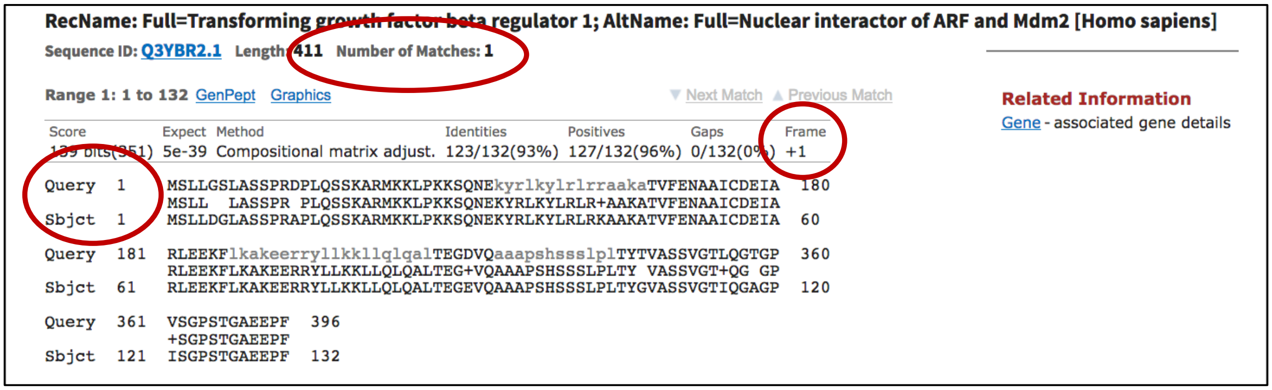

Before settling on the start codon we tested above, we go through the same process of resetting the start codon (not shown) to investigate the feasibility of the start codon located within exon 4 that agrees with the start codon in several other transcripts (Fig. S19). The result is a shorter CDS with a BLASTX result that shows a frame shift (Fig. S20).

Figure S19. Result of resetting the translation start of XM_005226786.4-00002 to a modified start site in exon 4.

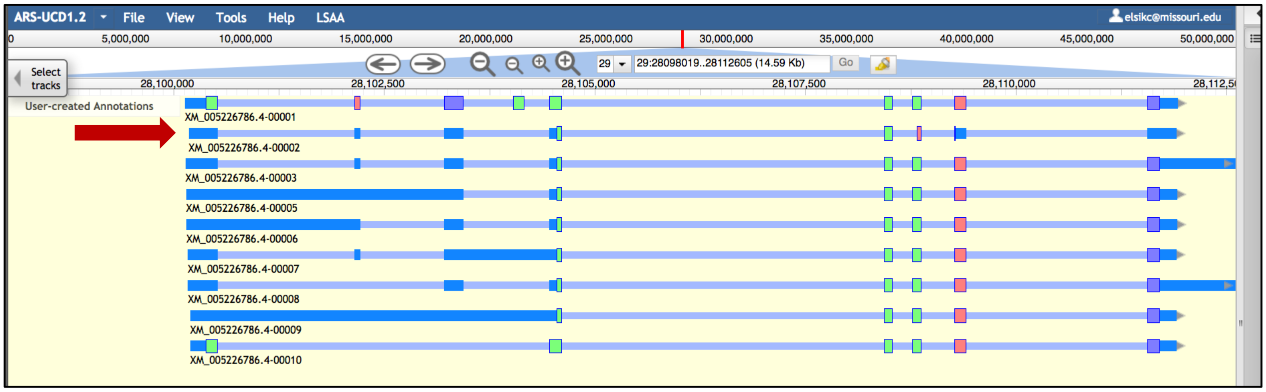

Figure S20. BLASTX alignment of CDS resulting from the start site modification in XM_005226786.4-00002 shown in Figure S17.

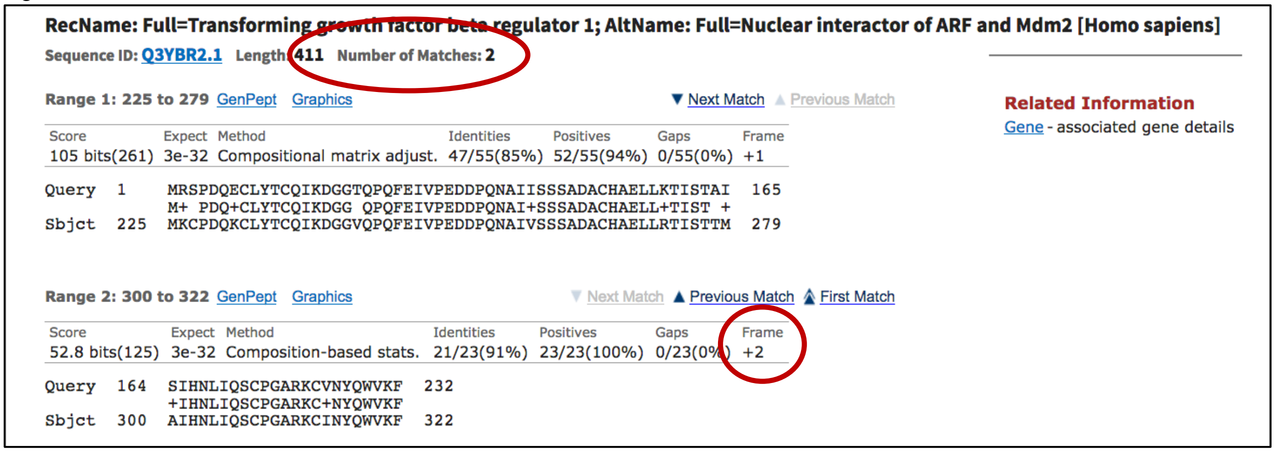

To further investigate the consequences of selecting different start codons, domain content of proteins are predicted by searching InterPro (https://www.ebi.ac.uk/interpro/search/sequence-search). First perform the search using the protein sequence of XM_005226786.4-00001 because it aligned well to the known protein (Fig S21).

Figure S21. Result of InterPro search with protein sequence encoded by XM_005226786.4-00001.

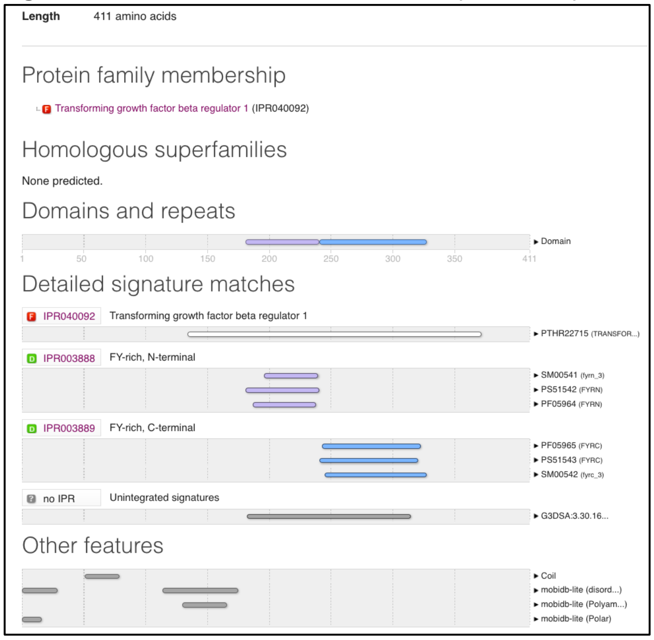

Then search InterPro using the three possible versions of XM_005226786.4-00002 (with original start codon, start codon in exon 1, and modified start codon within exon 4). Although the protein length for the version with a modified start codon in exon 4 is the shortest protein, it contains a signature match, a domain (FY-rich C-terminal) and unintegrated signature in common with XM_005226786.4-00001, while the version with the start codon in exon 1 had no domain matches (Fig. S22). Unfortunately, these results make it difficult to determine which start codon to select. Our final decision was the start codon in exon 1, which resulted in a 160 aa sequence, of which 132 aa aligned well with a known protein, while the version with the modified start codon in exon 4 was only 77 aa in length, of which only 55 aa aligned with the known protein.

Figure S22. InterPro results for proteins encoded by different possible CDS of XM_005226786.4-00002.

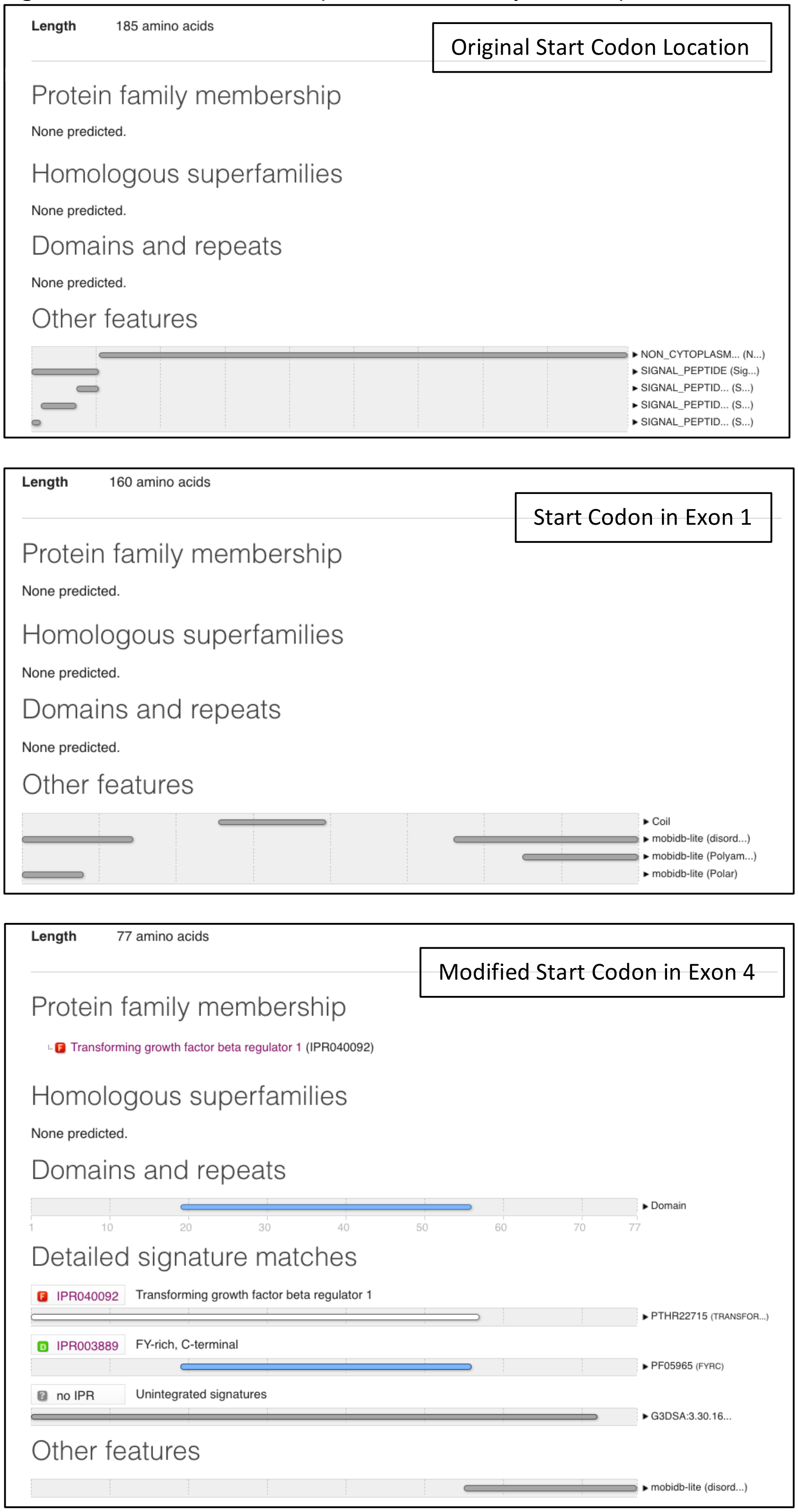

Of the remaining transcripts (XM_005226786.4-00003, -00005, -00006, -00007, -00008, -00009, -00010), there are only two alternative protein sequences, with most of the variation among the isoforms located in UTR. InterPro searches are done to check both of these proteins, showing that both proteins had domain matches (Fig. S23).

Figure S23. InterPro results for the two remaining CDS encoded by all the isoforms other than XM_005226786.4-00001 and XM_005226786.4-00002.

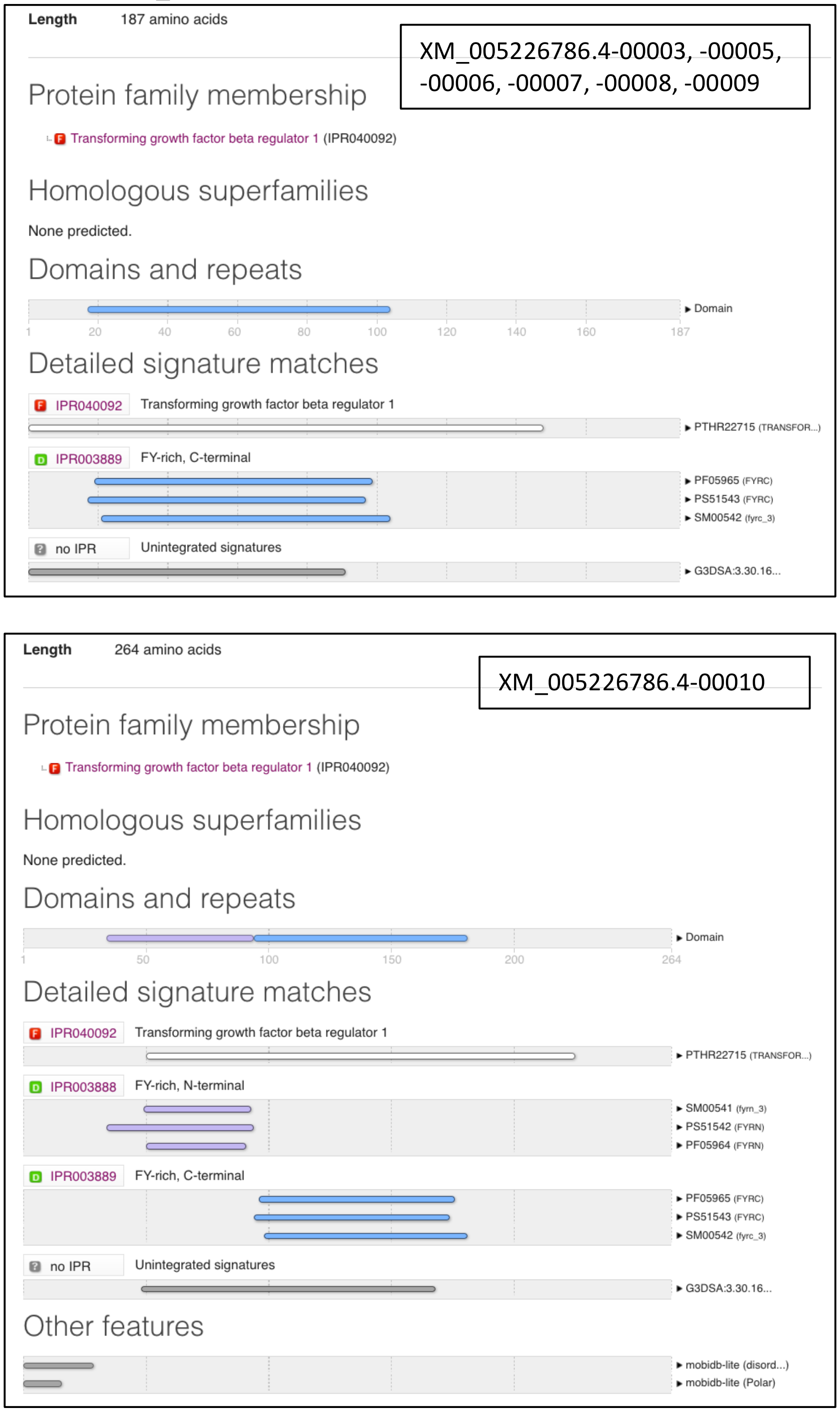

The next step is to determine whether RNA-seq tracks agree with the transcripts. First, remove the *Iso-Seq Combined PASA* track from the browser by clicking the X in the track label. Then go back to BovineMine to determine which RNA-seq tracks to view. Click on “EXPRESSION” in the template category bar. Select the “Bovine Transcript ID → Gene ID -> Expression” template. Enter the RefSeq transcript ID (XM_005226786.4), and click “Show Results”. The output shows that testis has the highest level of gene expression, but several other tissues also have sufficient levels (Fig. S24).

Figure S24. Process of using BovineMine to identify tissues in which the gene is highly expressed.

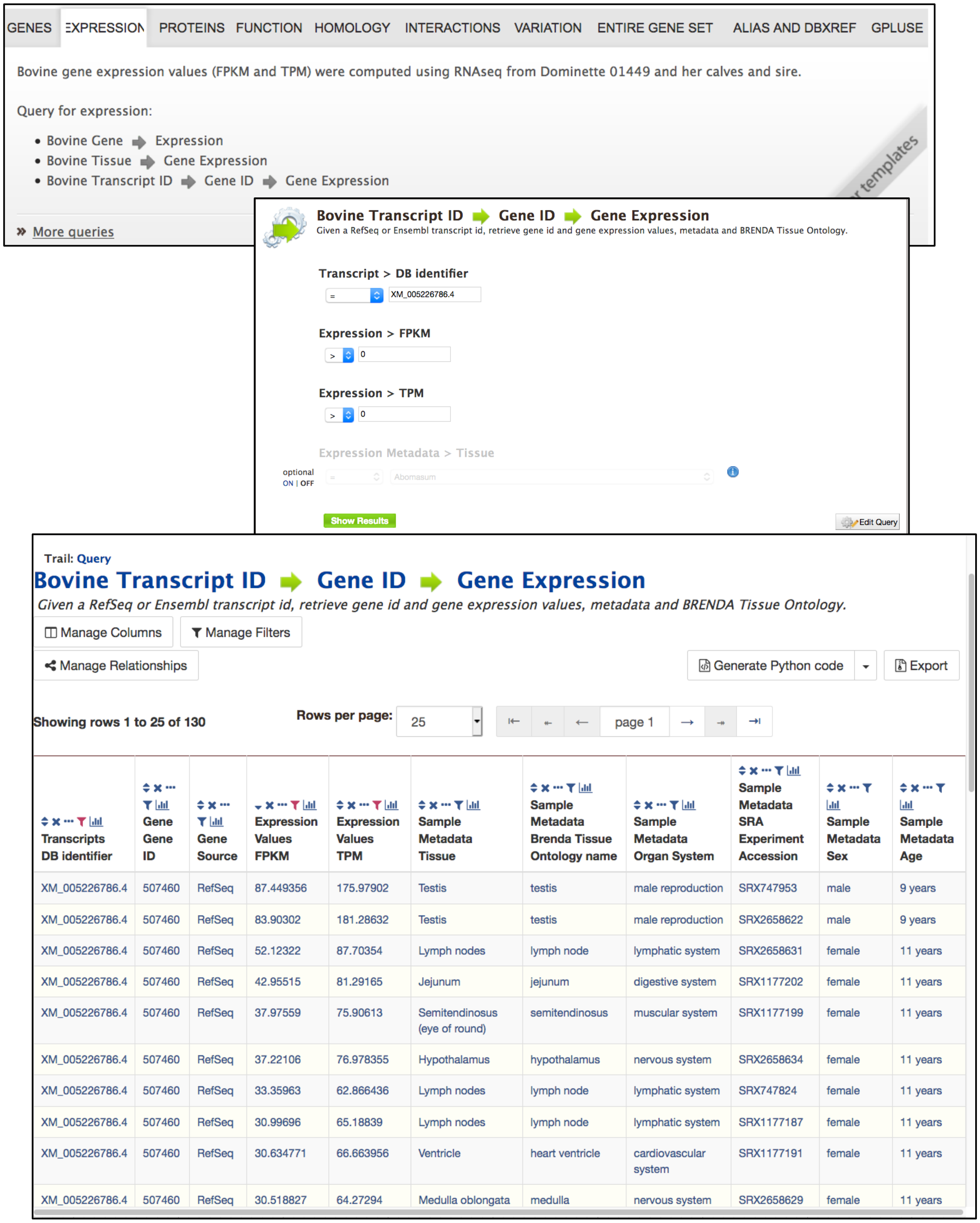

Transcripts DB identifier	Gene Gene ID	Gene Source	Expression Values FPKM	Expression Values TPM	Sample Metadata Tissue	Sample Metadata Brenda Tissue Ontology name	Sample Metadata Organ System	Sample Metadata SRA Experiment Accession	Sample Metadata Sex	Sample Metadata Age
XM_005226786.4	507460	RefSeq	87.449356	175.97902	Testis	testis	male reproduction	SRX747953	male	9 years
XM_005226786.4	507460	RefSeq	83.90302	181.28632	Testis	testis	male reproduction	SRX2658622	male	9 years
XM_005226786.4	507460	RefSeq	52.12322	87.70354	Lymph nodes	lymph node	lymphatic system	SRX2658631	female	11 years
XM_005226786.4	507460	RefSeq	42.95515	81.29165	Jejunum	jejunum	digestive system	SRX1177202	female	11 years
XM_005226786.4	507460	RefSeq	37.97559	75.90613	Semitendinosus (eye of round)	semitendinosus	muscular system	SRX1177199	female	11 years
XM_005226786.4	507460	RefSeq	37.22106	76.978355	Hypothalamus	hypothalamus	nervous system	SRX2658634	female	11 years
XM_005226786.4	507460	RefSeq	33.35963	62.866436	Lymph nodes	lymph node	lymphatic system	SRX747824	female	11 years
XM_005226786.4	507460	RefSeq	30.99696	65.18839	Lymph nodes	lymph node	lymphatic system	SRX1177187	female	11 years
XM_005226786.4	507460	RefSeq	30.634771	66.663956	Ventricle	heart ventricle	cardiovascular system	SRX1177191	female	11 years
XM_005226786.4	507460	RefSeq	30.518827	64.27294	Medulla oblongata	medulla	nervous system	SRX2658629	female	11 years

Go to the Faceted Track Selector, filter for RNA-seq PE Junctions (arcs) tracks and enter “Testis” name in the search box, and select the track. Repeat the search for other tissues shown to have high expression levels in the BovineMine search, such as Lymph Nodes and Jejunum (Fig. S25).

Figure S25. Using the Faceted Track Selector to select RNASeq PE Junctions (arcs) tracks.

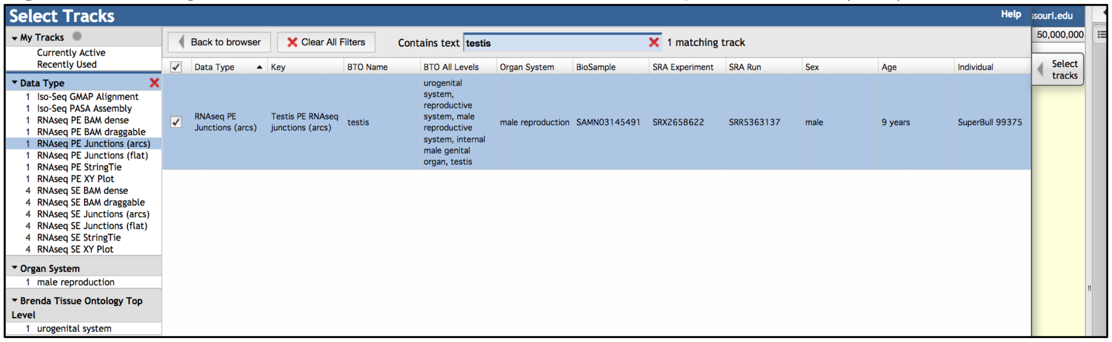

The junction arc tracks allow you to quickly determine whether RNA-seq supports a splice junction. The arcs represent connections within spliced read alignments. The thickness of each arc represents the number of reads (Fig. S26).

Figure S26. Apollo view showing RNAseq PEJunctions (arcs) tracks for three tissues.

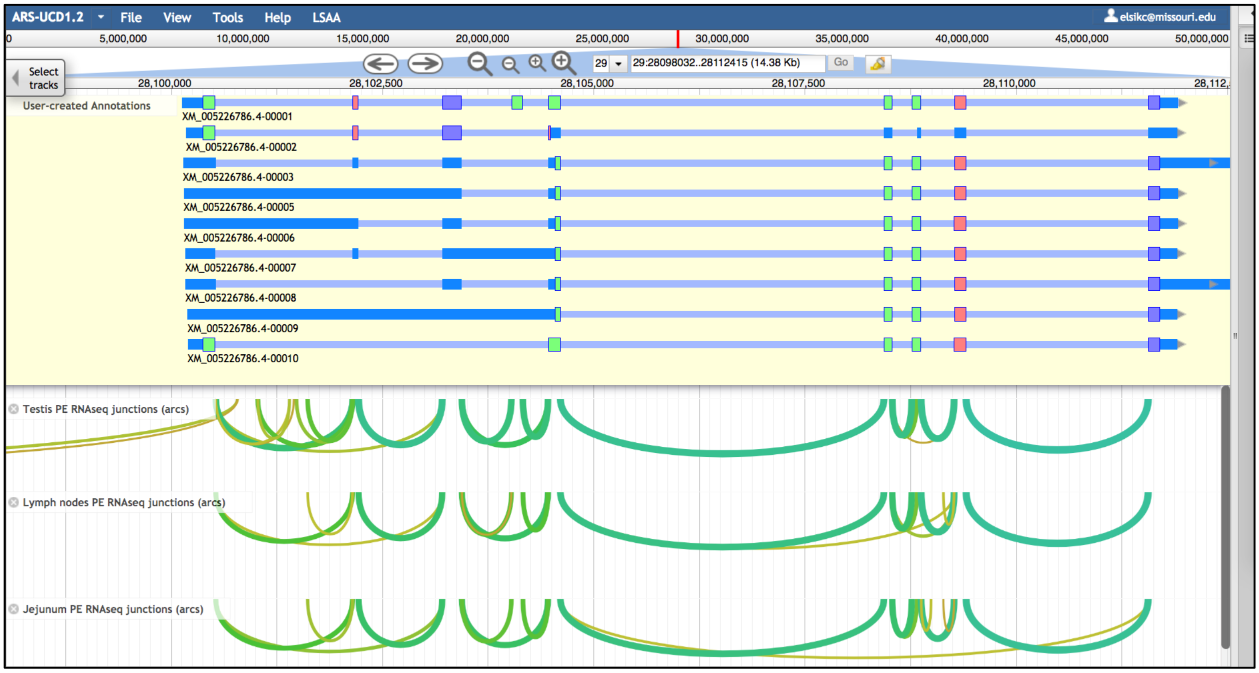

We will further investigate XM_005226786.4-00002, in which exon 6 is smaller than the corresponding exon in the other transcripts. Zooming in shows that there may be RNA-seq support for the shorter exon (Fig. S27).

Figure S27. Zoomed in view comparing junctions to exon edges. Light green junctions appear to support the larger intron in XM_005226786.4-00002.

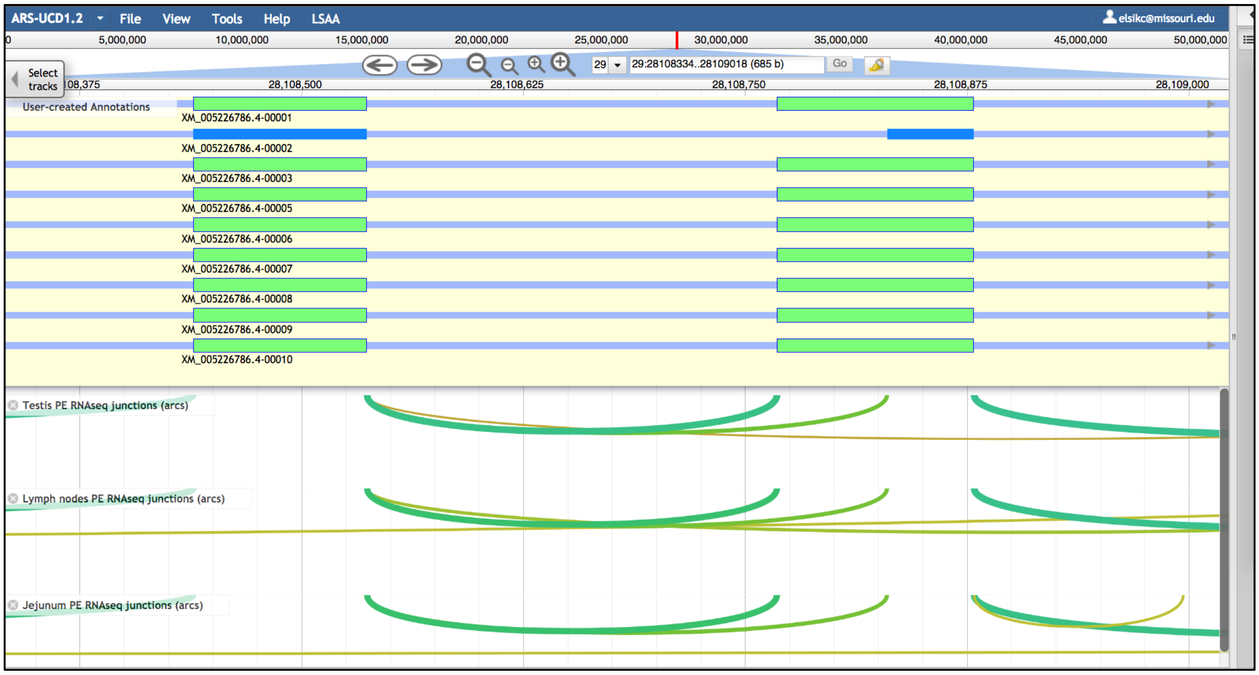

The *PE RNAseq junctions (arcs)* tracks are removed by clicking the X in the track labels, and *PE RNAseq junctions (flat)* tracks shown. The junction highlighted in red in the *Testis PE RNAseq junctions (flat)* track seems to match the questionable intron in XM_005226786.4-00002 (Fig. S28).

Figure S28. *RNAseq PE junctions (arc)* tracks have been replaced with *RNAseq PE junctions (flat)* tracks. The outlined junction appears to support the intron of XM_005226786.4-00002.

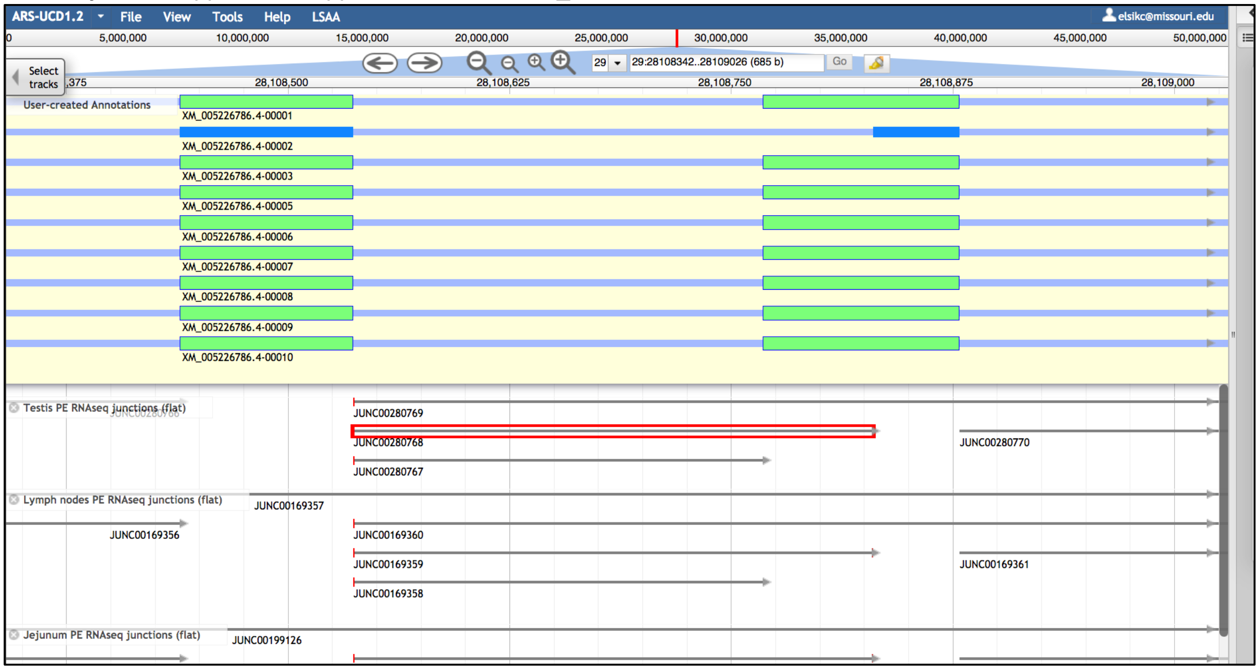

Right clicking a flat junction allows you to view details. The “Score” is the number of reads supporting this intron. The fact that there are only 12 reads shows that this is a lowly expressed transcript variant (Fig. S29).

Figure S29. Details about the selected junction. The “Score” is the number of reads with the same junction.

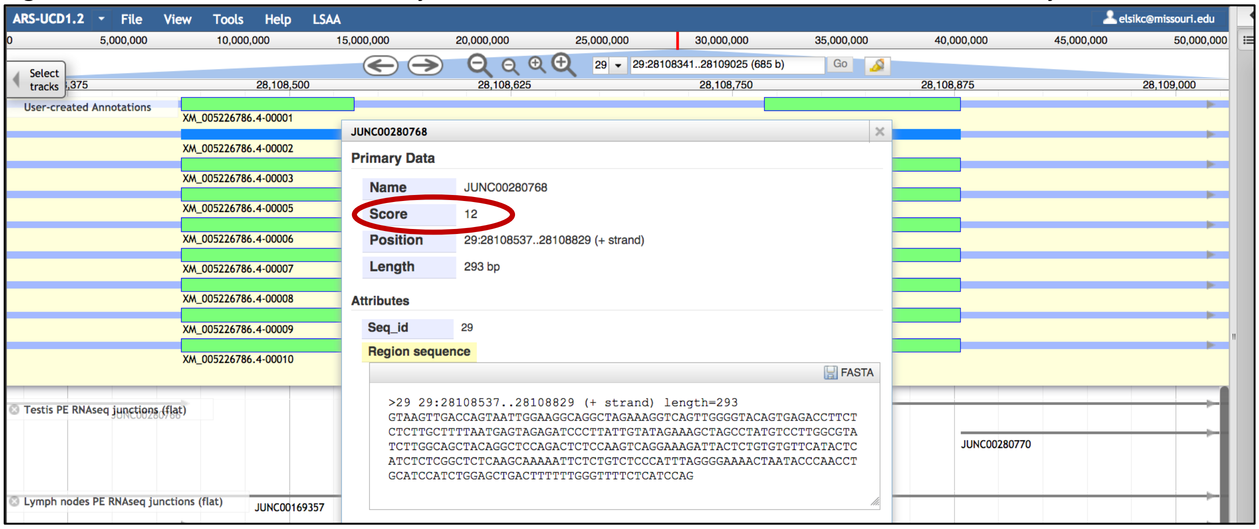

The *PE RNAseq junctions* (flat) tracks are removed by clicking the X in the track labels, and the track selector is used to select the *Testis PE BAM draggable* track, which shows individual RNA-seq read alignments. The aligned parts of the reads are the darker red and blue regions, and connections between parts of spliced reads are shown in lighter red and blue. In the figure below, the BAM track has been configured by right clicking the label and selecting “Hide unspliced reads”. Four of the reads in this view match the questionable intron. Clicking a read outlines it in red and also highlights exon edges in all other tracks that match the read’s splice junctions. Fig. S30 shows red edges on the boundaries of the exons in the questionable transcript. Thus, this transcript is supported by RNA-seq.

Figure S30. Apollo view showing spliced RNA-seq read alignments in the *Testis PE BAM draggable* track, zoomed in to visualize agreement of read splice sites with the questionable exon in XM_005226786.4-00002. Clicking a single read alignment highlights it in red. The red edges of the questionable splice site in XM_005226786.4-00002 indicate agreement with the RNA-seq read.

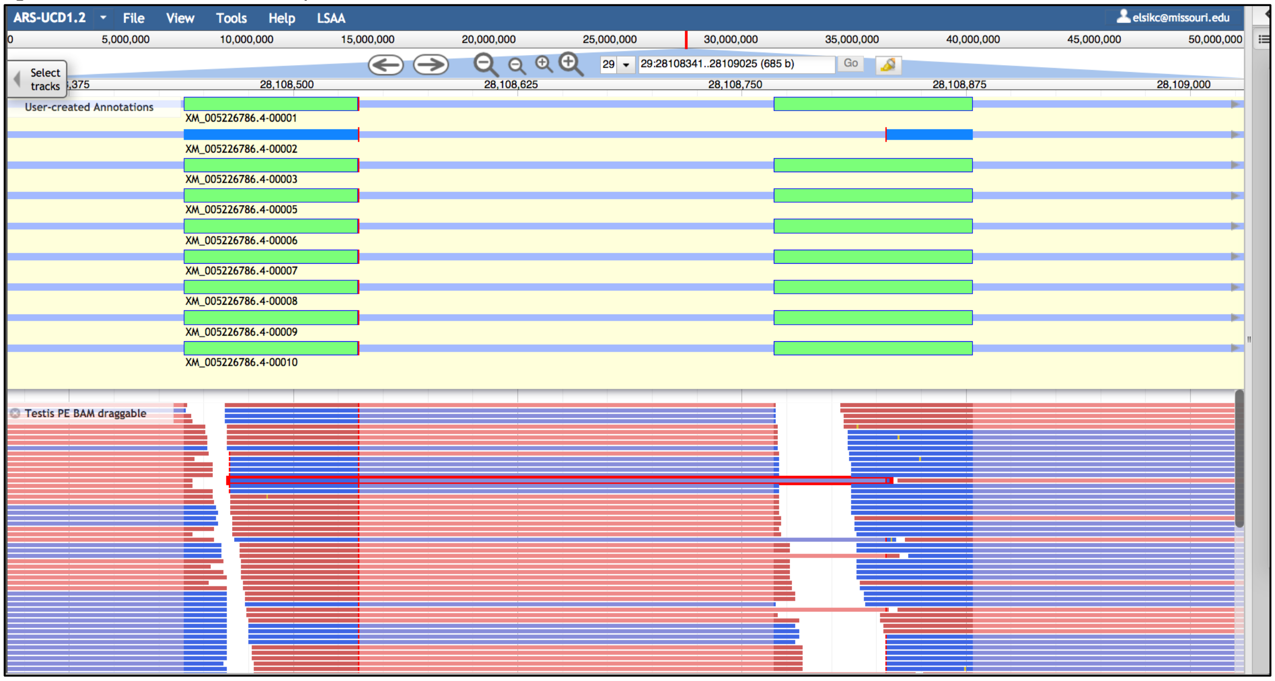

At this point we have finished evaluating the transcripts. To summarize, we added 8 new transcript isoforms to this locus. Most of the new isoforms varied only in the 5’ UTR, with only two new protein isoforms. The most challenging part of this annotation was determining the correct position of the translation start site for a transcript XM_005226786.4-00002 that was already present in the RefSeq dataset (original id NM_001075315.1). None of the potential CDS were well supported when considering both the BLASTX and InterPro results. One may question the validity of the exon/intron structure. However, two pieces of evidence support the structure. First, there is RNA-seq support (albeit low) in multiple tissues for the questionable exon. Second, the original RefSeq identifier begins with “NM”, and transcripts with “NM” ids are considered to have better support than those with “XM” ids. The GenBank report at NCBI (https://www.ncbi.nlm.nih.gov/nuccore/NM_001075315.1) shows that this transcript sequence was derived from a submitted sequence (BT026212) from a full-length cDNA sequencing project. On the other hand, the report also indicates that NM_001075315.1 is a “PROVISIONAL REFSEQ” and has not been subject to further review. Our decision is to keep this transcript, use the CDS with the best alignment to the known protein (with the start codon in exon 1), and add comments about it in the Information Editor.

To open the Information Editor, hold the alt key and click any of the transcripts. On the left side of the Information Editor is information about the gene. Note that the gene name is prefilled and should not be changed. At BGD we use the automatically entered gene names as unique identifiers and they are temporary. Instead, you can enter the gene symbol and/or gene description. On the left side of the panel is information for an individual transcript, which can be selected in the pulldown menu at the top left of the Information Editor. We select XM_005226786.4-00002. In the DBXref section, then enter the original RefSeq identifier under “Accession” and “RefSeq” under DB (Fig. S31). Scroll down to comments and add detailed comments about the uncertainty of the translation start and CDS (Fig. S32).

Figure S31. The upper part of the Information Editor. Entering information (e.g. gene symbol and description) for the gene on the left fills in the same information for all transcripts. The pulldown menu at the top is used to select individual transcripts to enter more specific information on the right. Note that the original RefSeq id was added as a DBXRef for XM_005226786.4-00002.

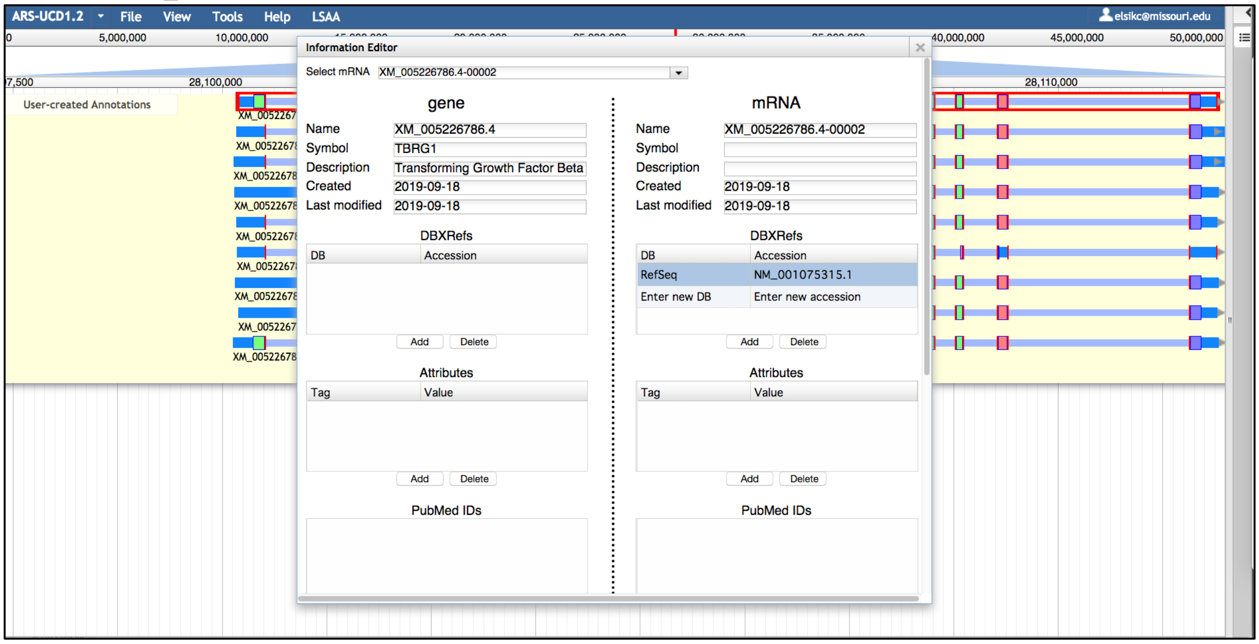

Figure 32. The lower part of the Information Editor, with a comment added about transcript XM_005226786.4-00002.

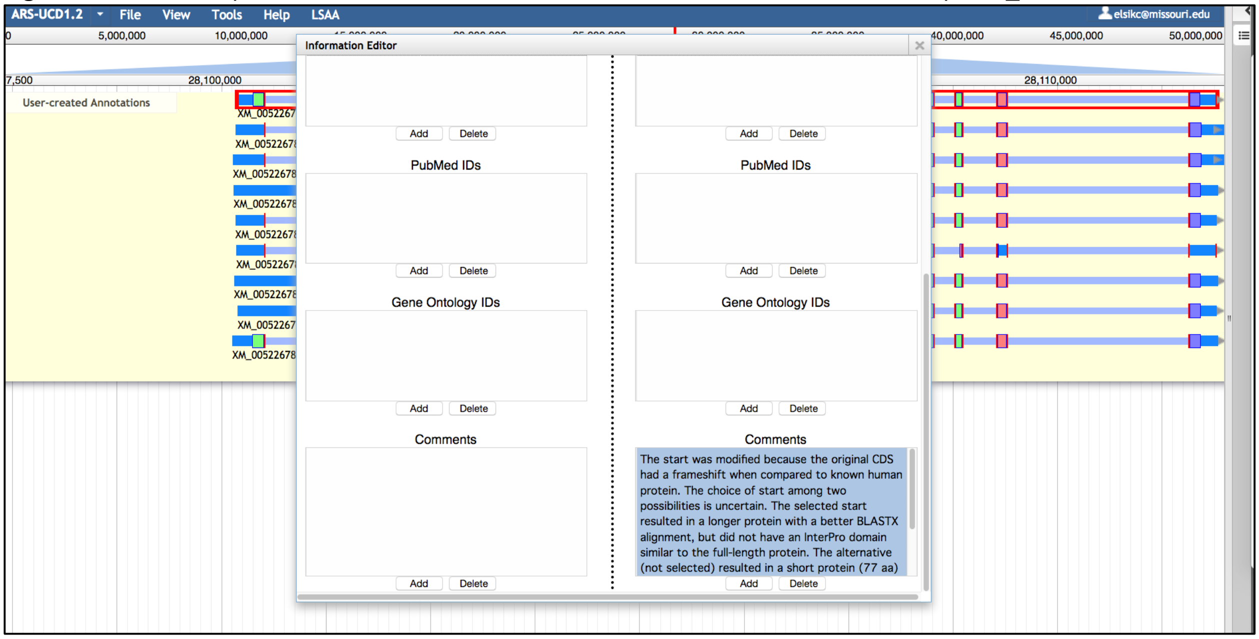

Supplement: Supplementary file 3 — Appendix S1 Annotation example 1. [file AGE-51-675-s003.pdf]
