# Supplementary material for: Using online tools at the Bovine Genome Database to manually annotate genes in the new reference genome
Source: Anim Genet. 2020 Jun 14;51(5):675–82. doi: 10.1111/age.12962 (PMC7540445; doi:10.1111/age.12962)
Supplement: Supplementary file 4 — Appendix S2 Annotation example 2. [file AGE-51-675-s004.pdf]

APPENDIX S2 – ANNOTATION EXAMPLE 2

Read Example 1 prior to reading this example, because this example will omit some details on navigating, selecting tracks and using Apollo menus. This example shows the resolution of a split/merge disagreement between Ensembl and RefSeq gene models, addition of isoforms, and investigation of non-canonical splice sites. This particular issue was identified by scanning the *Gene Prediction Problems* tracks in JBrowse (Fig. S33).

Figure S33. Selecting the *Ensembl Protein Coding Split/Merge* and *RefSeq Protein Coding Split/Merge* tracks after highlighting the Gene Prediction Problems data type on the left.

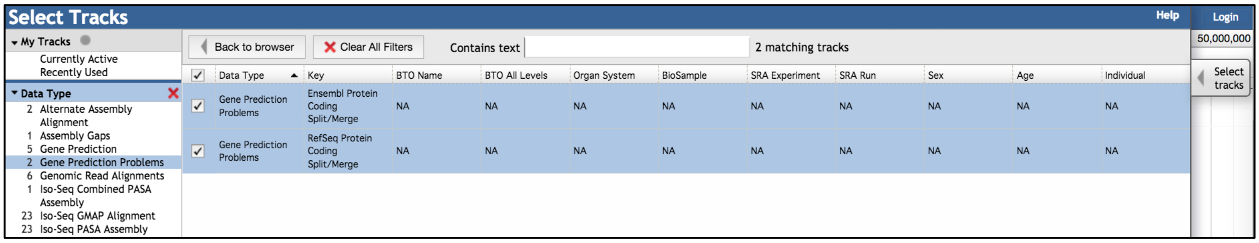

If you would like to do this example, follow the instructions in Example 1 to login to the Bovine Apollo Demo.

Use the Faceted Track Selector to select both *Gene Prediction Problems* tracks (Fig. S33). After going back to the browser, track labels will be visible on the left, but you may not see any genes at first. Enter "XM_002693872.6" into the navigation search box and click "Go" to access the region of interest. Notice that the gene in the *RefSeq Protein Coding Gene* track is exactly the same as the *RefSeq Protein Coding Split/Merge* track. This is because each Split/Merge track is simply the subset of genes that disagree with the alternative gene set. You can remove the *RefSeq Protein Coding Gene* track by clicking the X in the track label. Figure S34 shows the disagreeing genes.

Figure S34. The locus used in this example. RefSeq shows one gene where Ensembl shows two, one of which has two transcripts.

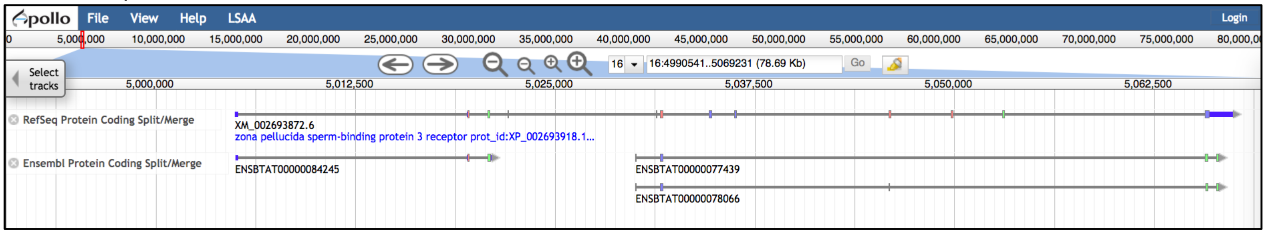

Use the Faceted Track Selector to select the *Iso-Seq Combined PASA* track. Notice that there are two spliced transcripts with the code "JE", for jejunum (Fig. S35).

Figure S35. Apollo view after the addition of the Iso-Seq Combined PASA Assembly track.

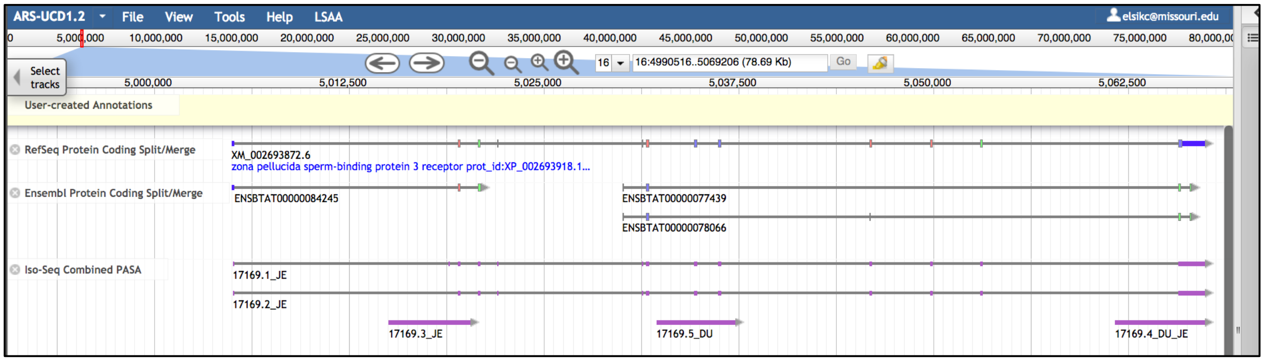

Use the Faceted Track Selector to select the *Jejunum Iso-Seq GMAP* track after filtering for *Iso-Seq GMAP Alignment* in the left panel. The GMAP alignment shows several spliced alignments, some of which were merged in the PASA assembly step (Fig. S36). The existence of unassembled Iso-Seq alignments that span the gene locus in question supports the single RefSeq gene locus rather than the two separate Ensembl gene loci. Now that we have support for the RefSeq transcript, start the annotation by dragging the RefSeq transcript to the Editing Area.

Figure S36. Apollo view after the addition of the Jejunum Iso-Seq GMAP track.

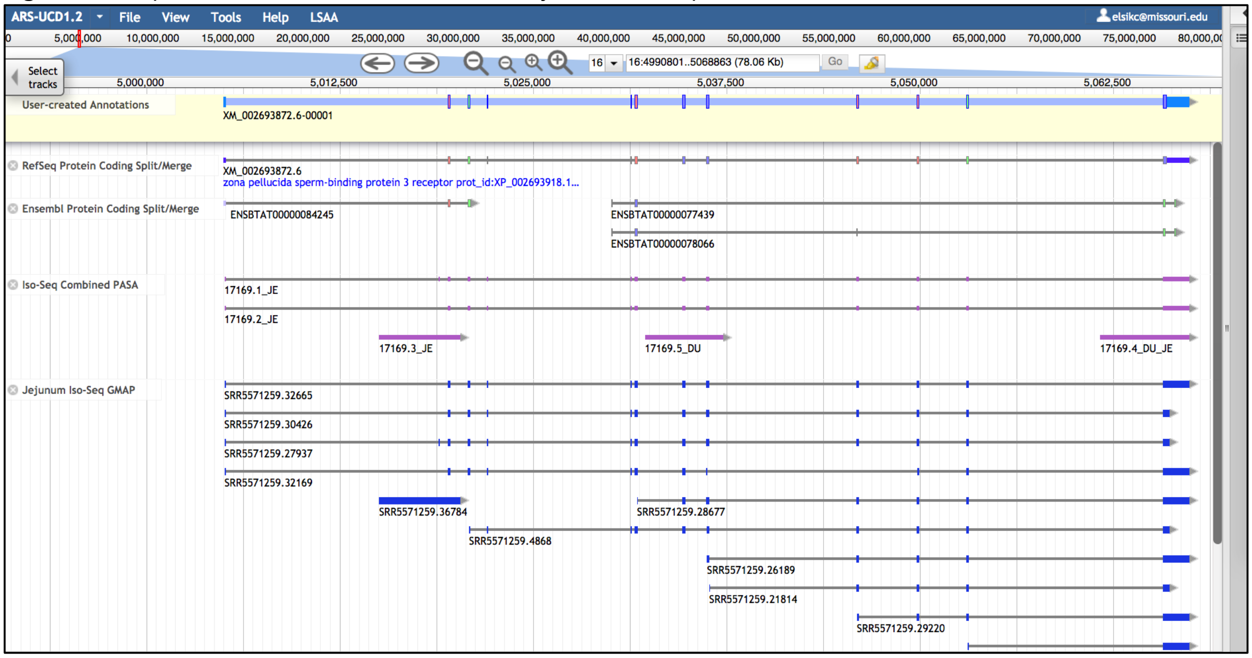

The *Jejunum Iso-Seq GMAP* track suggests new isoforms. Drag the four longest Jejunum Iso-Seq GMAP alignments to the Editing Area for further inspection (Fig. S37).

Figure S37. Apollo view after dragging the longest Jejunum Iso-Seq GMAP alignments to the Editing Area.

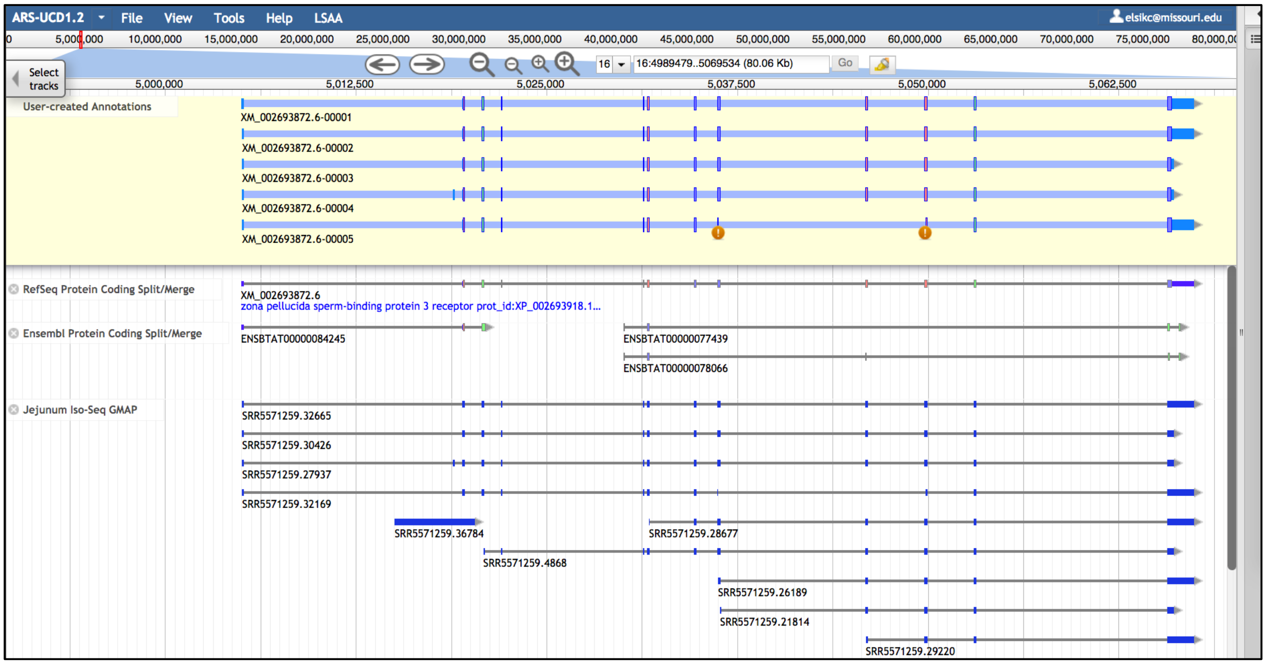

Investigate each transcript in the Editing Area by clicking it to highlight matching exon boundaries and determine whether there are any identical transcripts. Delete the redundant transcripts. We found XM_002693872.6-00001, -00002, and -00003 to be identical, so deleted -00002 and -00003 (Fig. S38).

Figure S38. View of Editing Area after deleting redundant transcripts in the Editing Area.

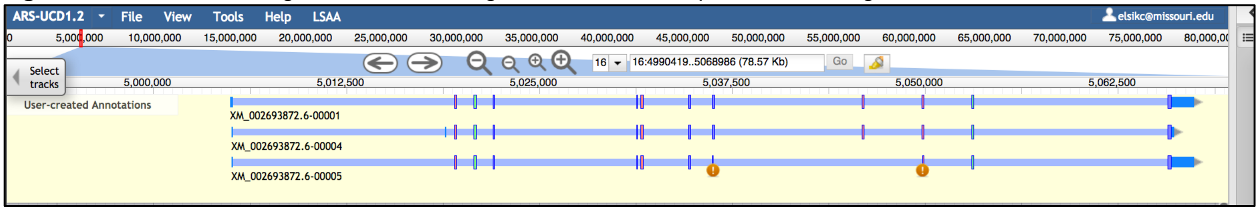

The next step is to check for further evidence that the locus contains one gene rather than two genes using RNA-seq. Go to BovineMine and enter XM_002693872.6 into the “Bovine Transcript ID → Gene ID → Gene Expression” template query to identify which tissues have high gene expression levels (described in Example 1). The output shows several tissues with high expression levels (Fig. S39).

Figure S39. Template query output.

Bovine Transcript ID ➡ Gene ID ➡ Gene Expression

Given a RefSeq or Ensembl transcript id, retrieve gene id and gene expression values, metadata and BRENDA Tissue Ontology.

Manage Columns | Manage Filters | Manage Relationships | Generate Python code | Export

Showing rows 1 to 25 of 52 **Rows per page:** 25 page 1

Transcripts DB identifier	Gene Gene ID	Gene Source	Expression Values FPKM	Expression Values TPM	Sample Metadata Tissue	Sample Metadata Brenda Tissue Ontology name	Sample Metadata Organ System	Sample Metadata SRA Experiment Accession	Sample Metadata Sex	Sample Metadata Age
XM_002693872.6	506707	RefSeq	830.9737	1441.8308	Duodenum	duodenum	digestive system	SRX747899	female	11 years
XM_002693872.6	506707	RefSeq	563.0691	1083.9614	Duodenum	duodenum	digestive system	SRX2658636	female	11 years
XM_002693872.6	506707	RefSeq	320.42706	606.79065	Spleen	spleen	"hemolymphoid system, immune system"	SRX1177200	female	11 years
XM_002693872.6	506707	RefSeq	316.4304	482.4161	Jejunum	jejunum	digestive system	SRX748031	female	11 years
XM_002693872.6	506707	RefSeq	197.24864	371.07104	Ascending colon	colon ascendens	digestive system	SRX747843	female	11 years
XM_002693872.6	506707	RefSeq	166.51816	315.07315	Ileum	ileum	digestive system	SRX747929	female	11 years
XM_002693872.6	506707	RefSeq	163.22061	310.4799	Descending colon	colon descendens	digestive system	SRX747849	female	11 years
XM_002693872.6	506707	RefSeq	152.94841	267.87704	Caecum	cecum	digestive system	SRX747865	female	11 years
XM_002693872.6	506707	RefSeq	104.87289	211.31258	Ileum	ileum	digestive system	SRX1177201	female	11 years
XM_002693872.6	506707	RefSeq	92.36649	168.78131	Bladder	urinary bladder	urinary system	SRX747813	female	11 years
XM_002693872.6	506707	RefSeq	47.70317	96.75506	Gall bladder	gall bladder	digestive system	SRX1177193	female	11 years
XM_002693872.6	506707	RefSeq	4.76926	9.498176	Liver	liver	digestive system	SRX747758	female	11 years

Back in Apollo, go to the Faceted Track Selector and use the filters on the left to select *RNAseq SE Junctions (arcs)* and *RNAseq PE Junctions (arcs)*. Use the search box on the top of the Faceted Track Selector to enter the tissues shown in the BovineMine query output, one at a time, and check the box for each track (duodenum, spleen, jejunum, ascending colon, ileum, caecum, bladder, gall bladder). This opens all of the selected arc tracks. In the browser, scroll down to view all of the arc tracks and remove the tracks that have little or no information. You will notice that for some tissues one track shows coverage (e.g spleen PE track and jejunum SE track) and the other (e.g. the spleen SE track and jejunum PE track) does not. This is due to differences in sequencing depth of the different libraries (Fig. S40).

Figure S40. Apollo views after selecting arc tracks. Three to four tracks are visible at one time. The scroll bar on the right of the browser allows scrolling down to view all the tracks, keeping the Editing Area at the top of the browser. The lower panel shows two tracks, *Spleen SE RNAseq Junctions (arcs)* and *Jejunum PE RNAseq Junctions (arcs)*, with little information for this gene, so they can be removed by clicking the X in the track labels.

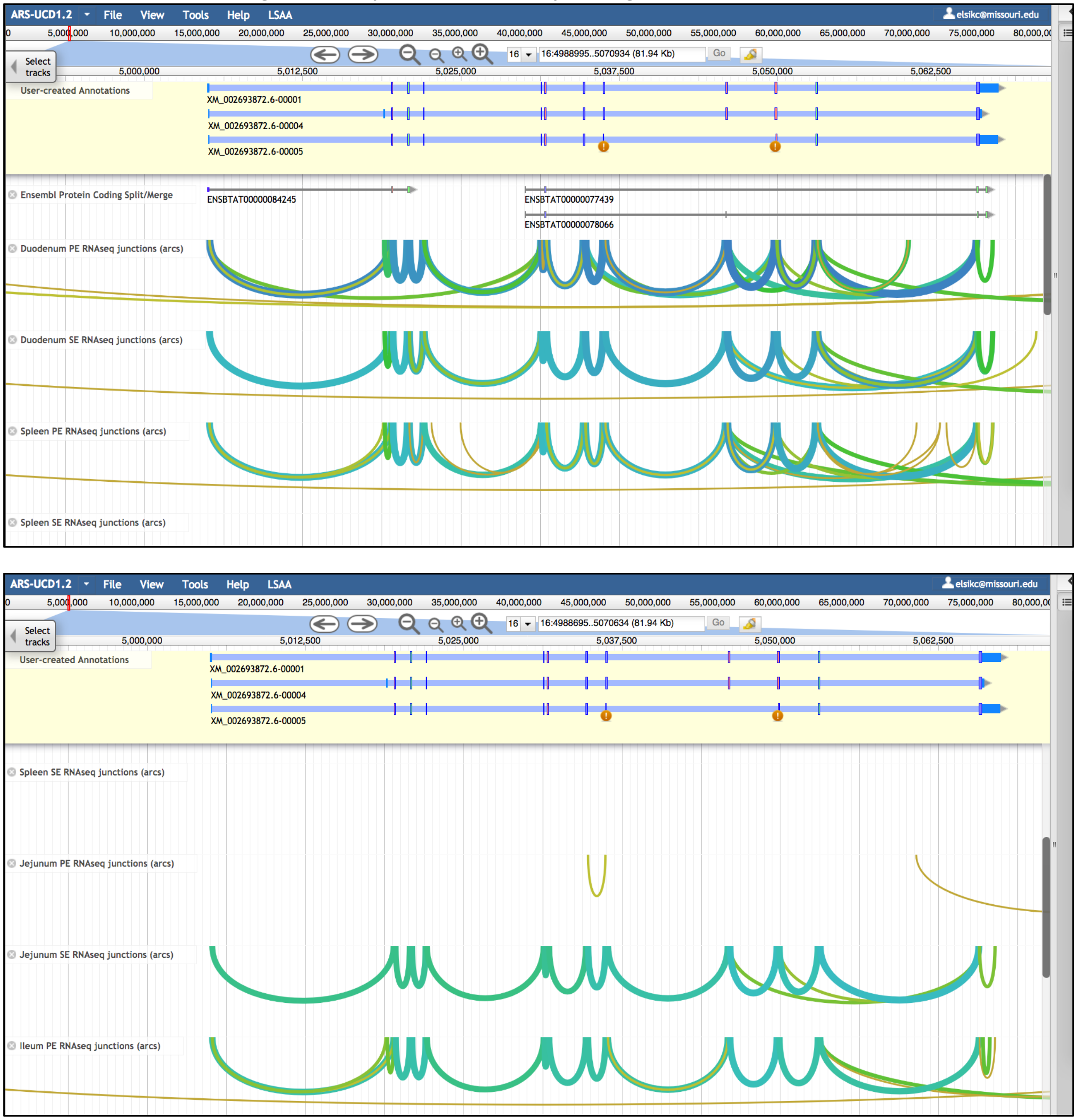

Zoom in to the intergenic region between the two Ensembl genes. A BAM draggable track will be used to further verify support for the connection between the exons of the RefSeq gene in this region. In Fig. S41, Duodenum SE and Jejunum SE seem to be good track choices, because they do not have alternative splice junctions in this region. After investigating both tracks we found the Jejunum SE track to be easier to view because its lower sequencing depth made it less computationally intensive for the browser.

Figure S41. Apollo view of arc tracks zoomed in to region of split/merge disagreement.

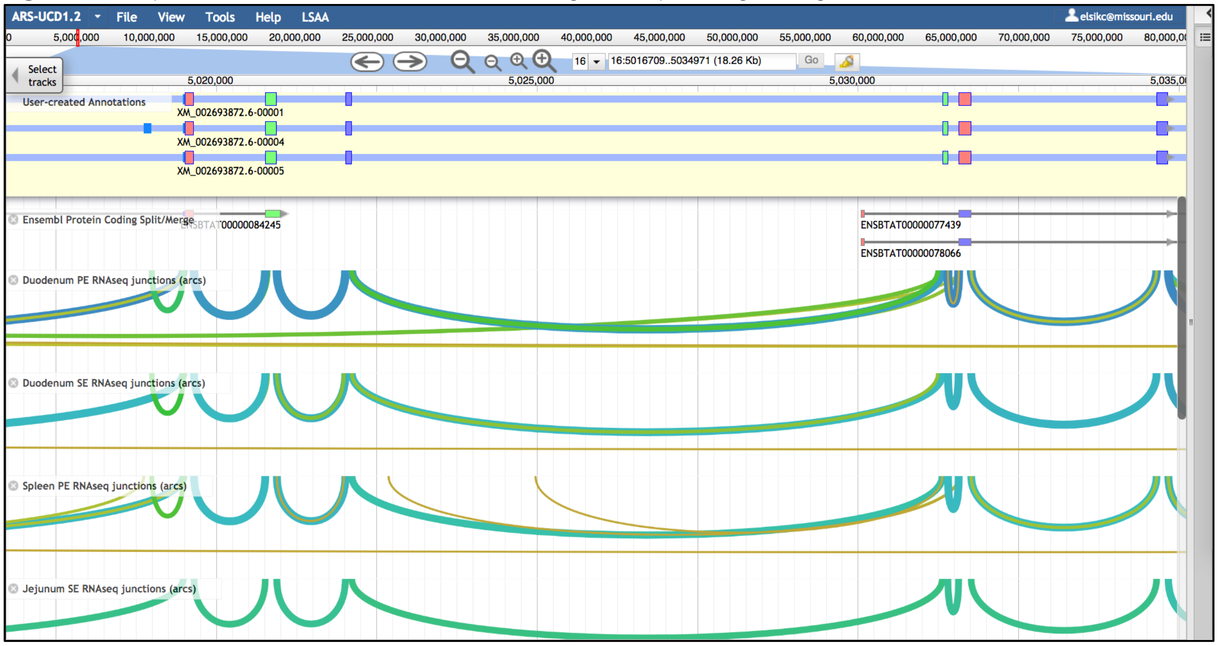

Remove all arc tracks except Jejunum SE and the use the Faceted Track Selector to open the *Jejunum SE BAM (draggable)* track. Right-click the track label and select “Hide unspliced reads”. The resulting view shows spliced read alignment support for the RefSeq gene model in the region of interest (Fig. S42).

Figure S42. Apollo view showing Jejunum SE BAM (draggable) track. Clicking a read makes matching edges barely visible.

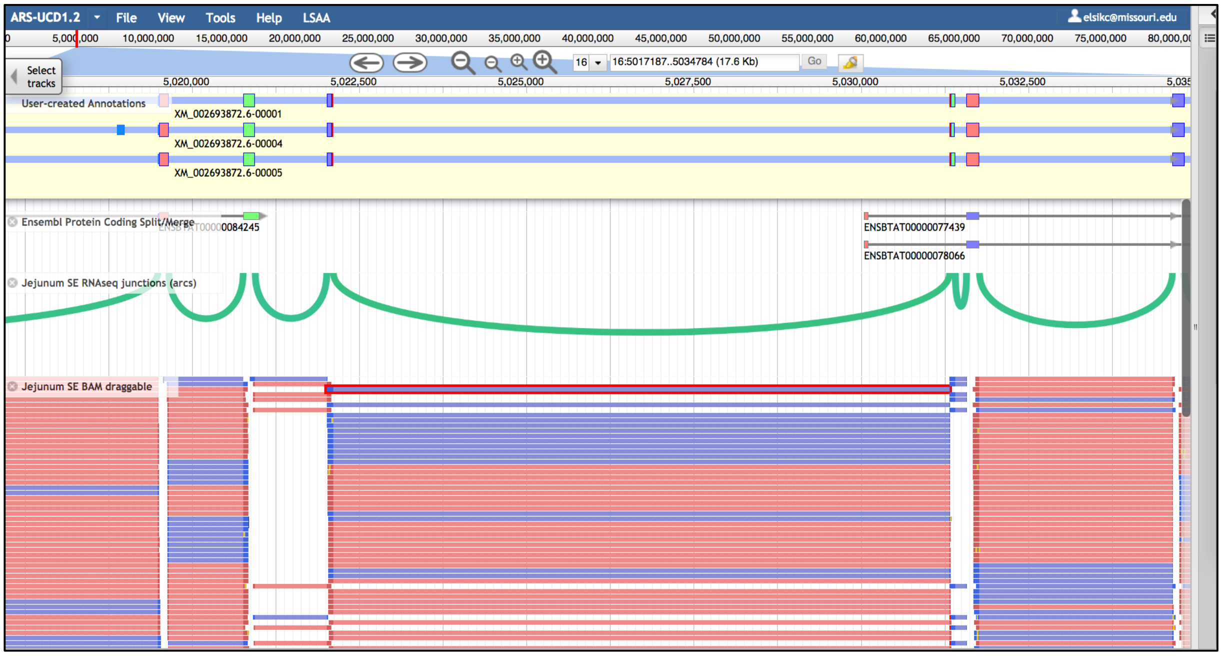

Exon edge matching to spliced reads is difficult to see when zoomed out too far, so zoom in to each splice site of interest to check for edge matching. Click an exon in the Editing Area and ensure that red edges are showing at both the exon boundary and at the splice boundaries in the spliced reads. Doing so at both ends of the large intron shows good RNA-seq read support for this intron (Figs. S43 and S44).

Figure S43. Zoomed in view at 5' end of large intron showing matching RNA-seq junctions.

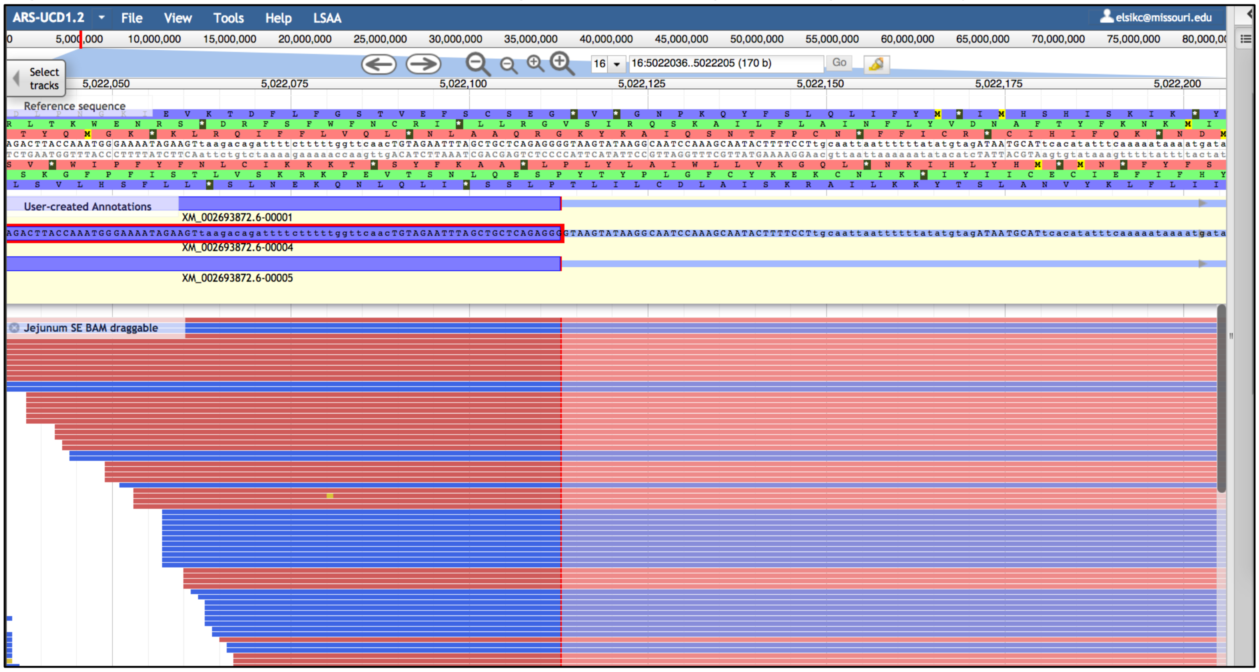

Figure S44. Zoomed in view at 3' end of large intron showing matching RNA-seq junctions.

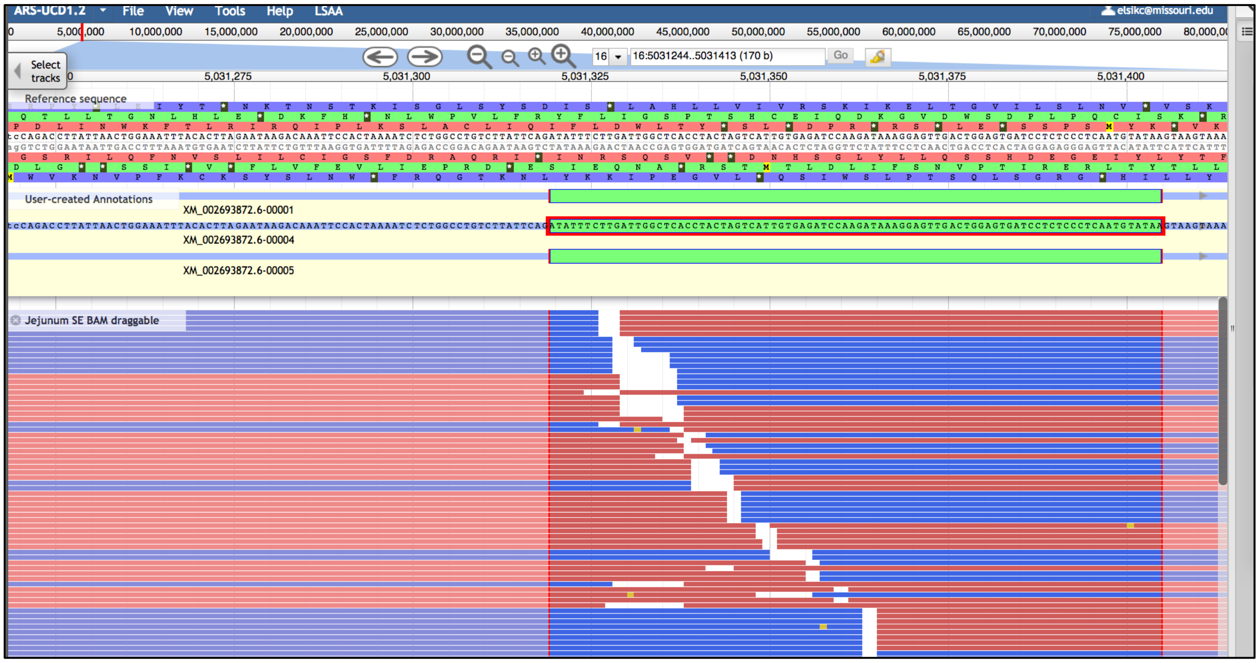

A similar investigation is performed on the smaller introns to the left and the right of the large intron to verify gene continuity. Navigate to the intron upstream of the large intron. The intron is small enough to allow viewing both adjacent exons at the same time, and edge matching can be checked by clicking one of the spanning reads (Fig. S45). Finally, check the intron downstream of the large intron to verify continuity on that side (Fig. S46). All introns that span the gap between the Ensembl genes are well-supported by RNA-seq.

Figure S45. Zoomed in view of small intron upstream of the large intron, showing matching RNA-seq junctions.

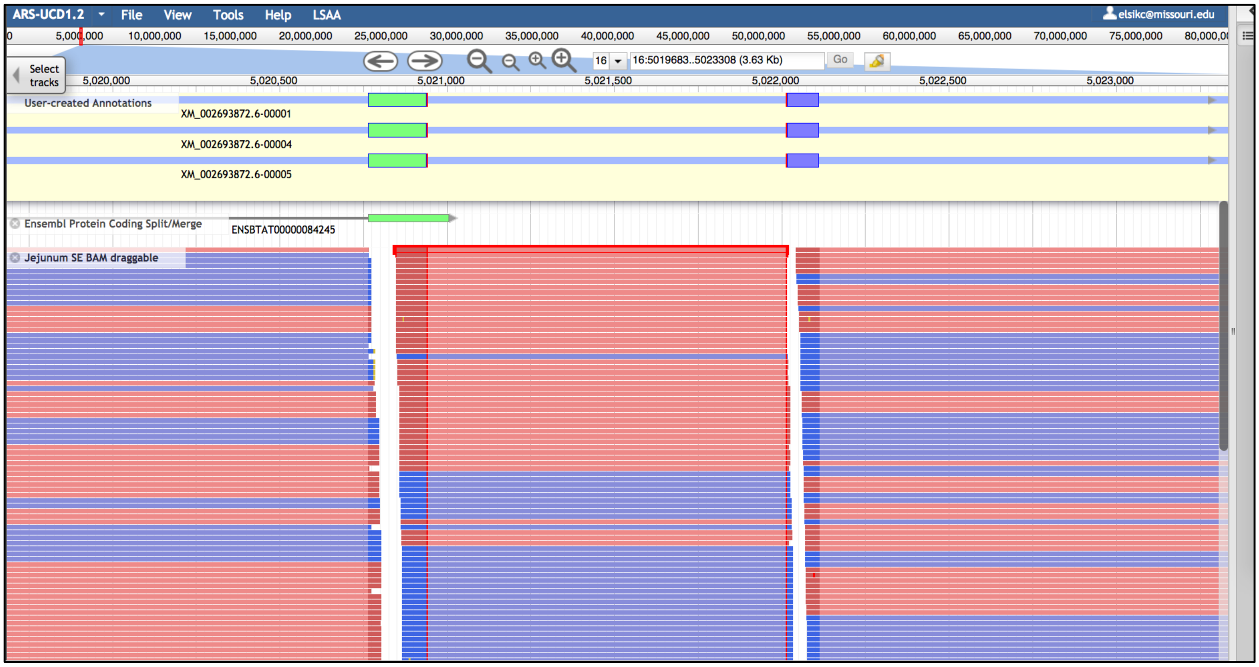

Figure S46. Zoomed in view of small intron downstream of the large intron, showing matching RNA-seq junctions.

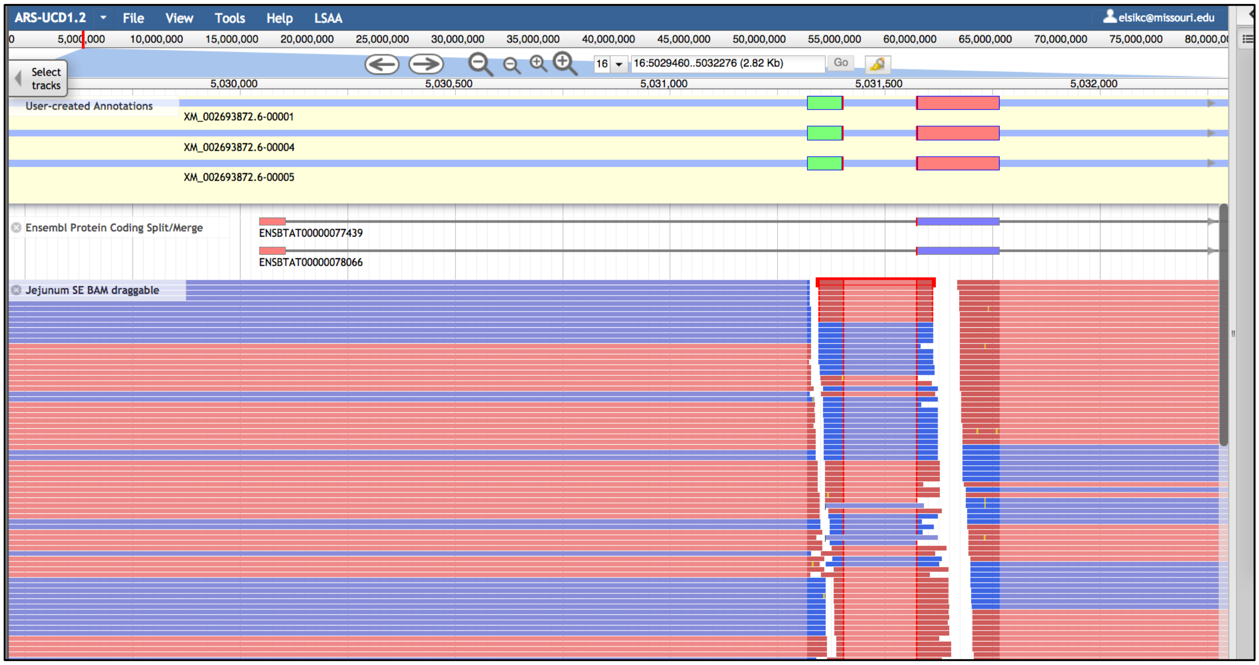

The investigation of the split/merge issue is complete, and has led to the conclusion that the RefSeq gene is correct.

The next task is to investigate isoform XM_002693872.6-00005, which has two exons that are shorter than their counterparts in the other transcripts, and have non-canonical splice sites, as shown with exclamation marks in the Editing Area (Fig. S47).

Figure S47. Zoomed in views of the non-canonical splice sites in XM_002693872.6-00005.

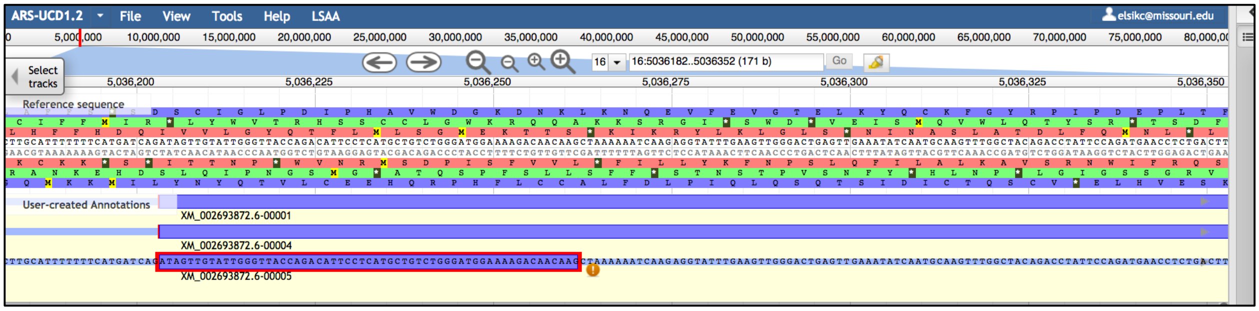

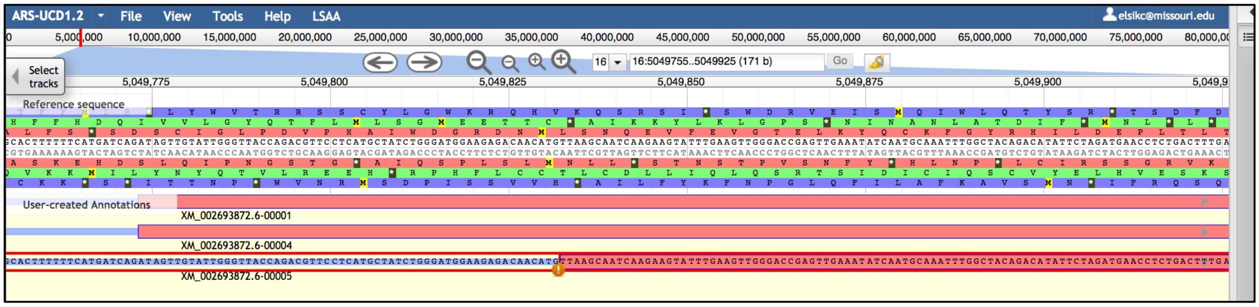

Remove the BAM track and use the Faceted Track Selector to bring back the highest depth *RNAseq junctions (arcs)* tracks (duodenum PE and SE, Spleen PE, Jejunum SE). A zoomed in view suggests that the *Duodenum PE RNAseq junctions (arc)* track may support the intron of interest (Fig. S48).

Figure S48. Apollo view with *RNAseq junctions (arcs)* tracks zoomed in to region of intron with non-canonical splice sites.

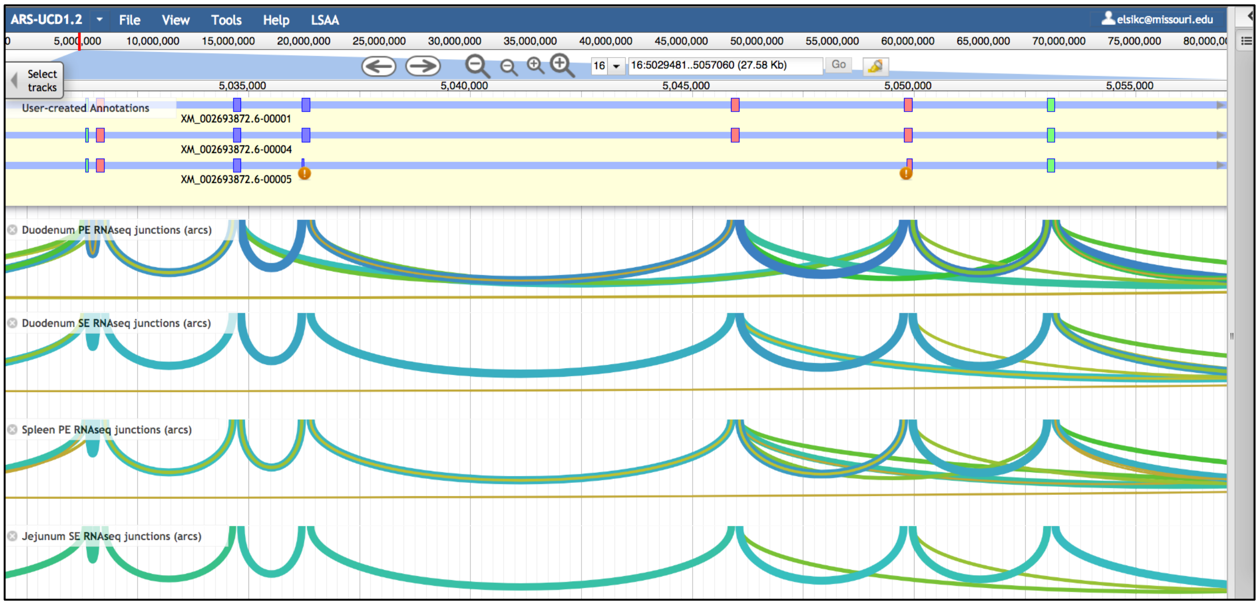

Remove the *RNAseq junctions (arcs)* tracks and use the Faceted Track Selector to select the *Duodenum PE RNAseq junctions (flat)* track. Fig. S49 shows a junction that appears to span the intron of interest.

Figure S49. Apollo view with highlighted RNA-seq junction that appears to support the intron of interest.

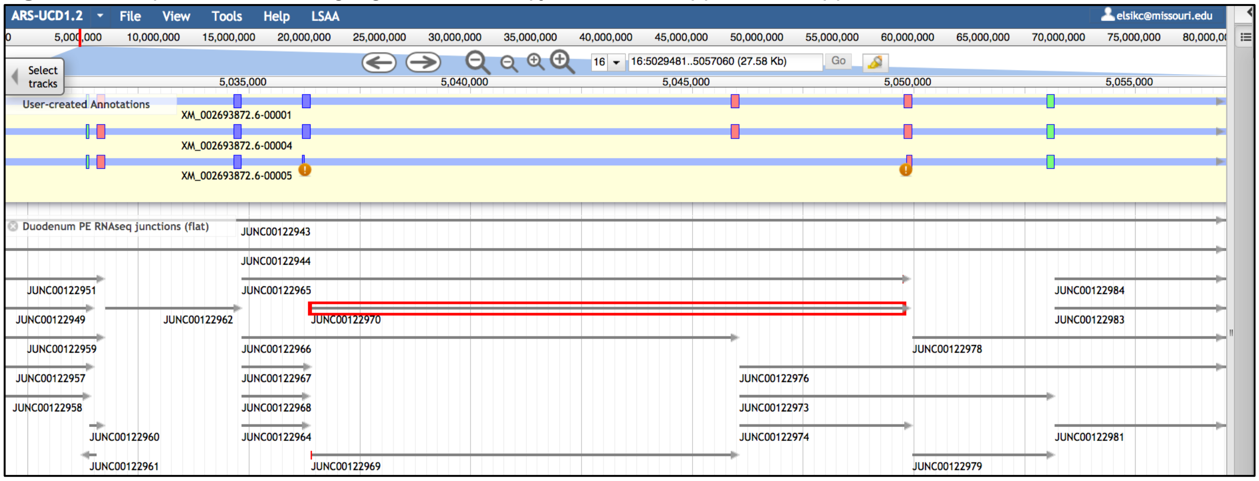

Right click the junction and click View Details for see more information. The “Score” represents the number of RNA-seq reads supporting the junction (Fig. S50). This junction is supported by 12 reads (a low level of RNA-seq support).

Figure S50. Information about the highlighted junction showing that 12 RNA-seq reads support this junction.

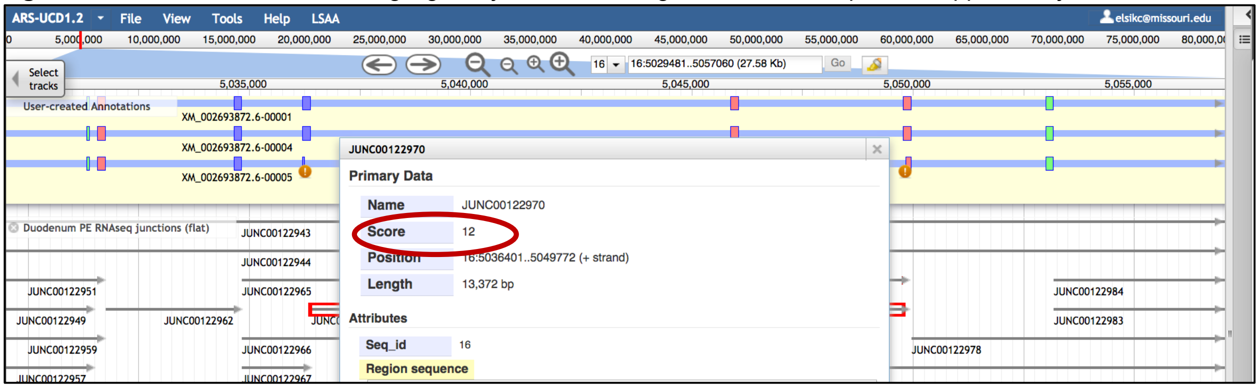

With the junction still highlighted, zoom in to each end of the intron to get a closer look. Figs. S51 and S52 show that this junction does not actually extend to the adjacent exons, and thus does not support this intron or the non-canonical splice sites.

Figure S51. Zoomed-in view at 5’ end of intron showing lack of RNA-seq support for the non-canonical splice site.

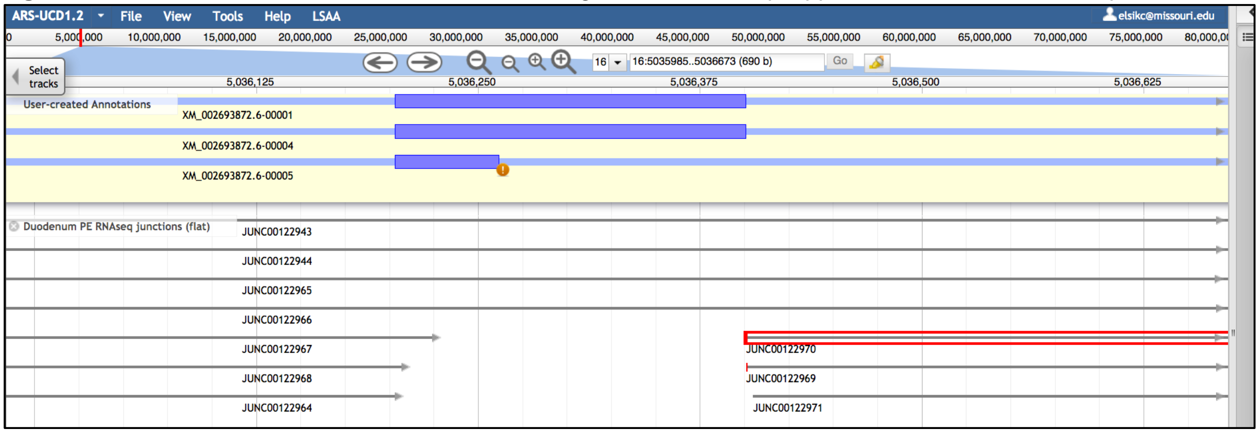

Figure S52. Zoomed-in view at 3’ end of intron showing lack of RNA-seq support for the non-canonical splice site.

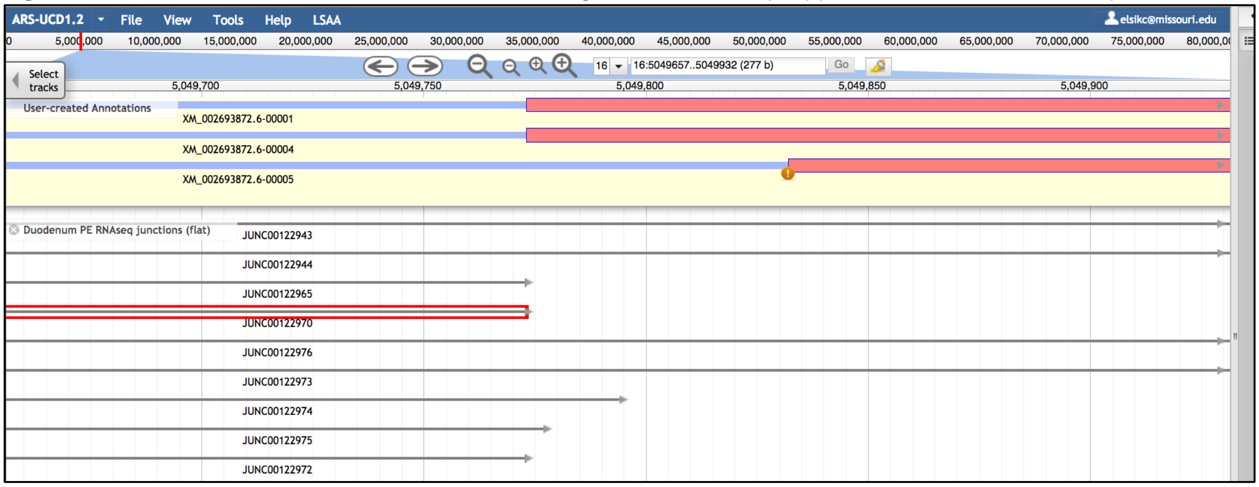

We can conclude that this intron with non-canonical splice sites does not have RNA-seq support. The next step is to investigate whether the non-canonical splice sites were due to poor a GMAP alignment. Remove the *Duodenum PE RNAseq junctions (flat)* track and use the Faceted Track Selector to open the *Jejunum Iso-Seq GMAP* track. By clicking the annotation in question (XM_002693872.6-00005) we can look at matching edges of the mapped transcripts and identify the transcript with the non-canonical splice sites to be SRR571259.32169. Clicking that transcript and selecting “View details” provides information about each of the exons in the “Subfeatures” section of the information panel (Figs. S53 and S54). Scroll down to look at the details for the 7th and 8th exons. The “Gap” attribute provides information about the alignment. For the 7th and 8th exons, “M59” and “M139” mean that the sequence had 59 and 139 matches, respectively (Fig. S54). There were no insertions (designated as “I”) or deletions (designated as “D”). The “Score” of each exon is 100, meaning that the aligned sequences were 100% identical (no substitutions). Furthermore, the “Target” information shows the coordinates of the alignments on the Iso-Seq sequence, and indicates that there was no gap in the Iso-Seq sequence between the alignment of the two exons. The surrounding exons also have perfect alignments. We conclude that this is a high quality GMAP alignment.

Figure S53. Right clicking SRR571259.32169 to view details.

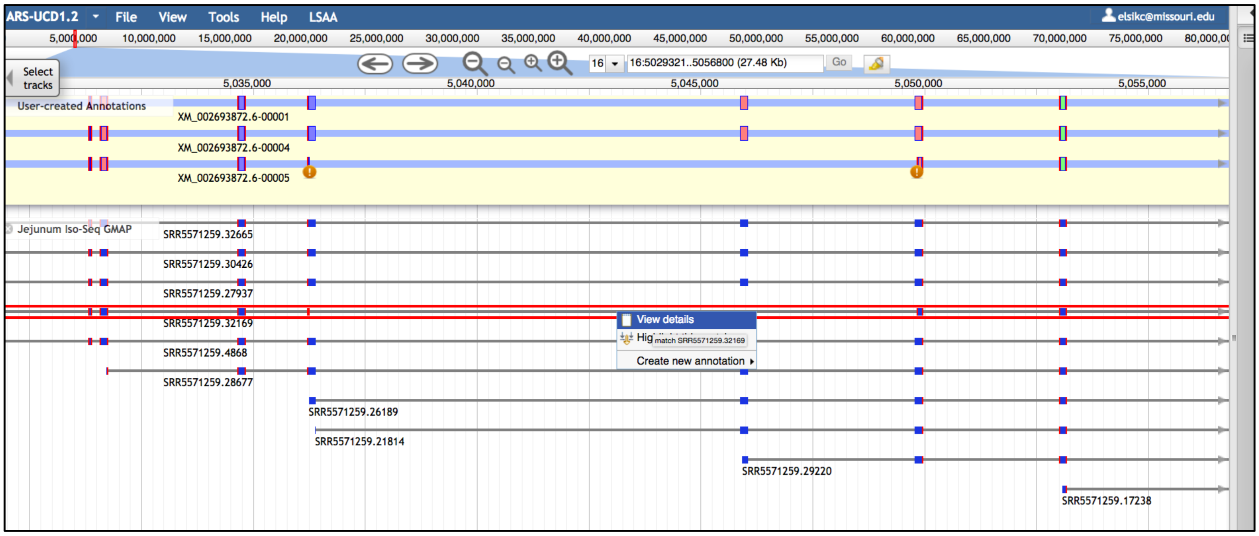

Figure S54. Information about the 7th and 8th exons in the information panel for the SRR571259.32169 alignment.

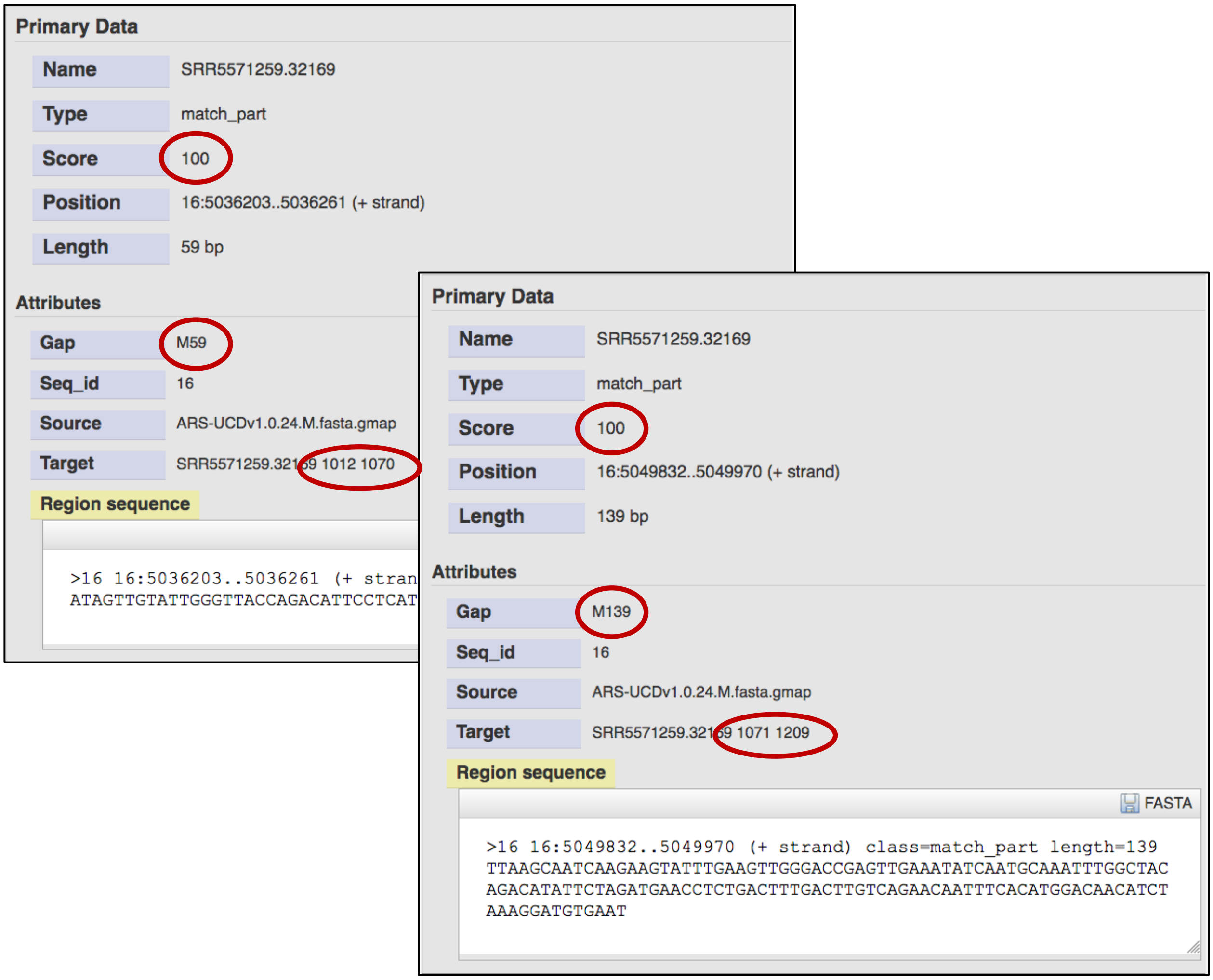

Given that we have a high quality Iso-seq alignment and that the affected exons are in the correct reading frame (same colors as the corresponding exons in the other transcripts), we believe this transcript to be real. One last check is to perform a BLASTX search of the coding sequence (CDS) of XM_002693872.6-00005 to a protein database. Although there are significant homologs in both the UniprotKB/Swiss-Prot and Model Organisms databases at NCBI, the percent identity is low, making it difficult to assess the alignment. Instead we try the Non-redundant protein sequences (nr) database, limiting the search to Mammalia (Fig. S55).

Figure S55. BLASTX search menu selecting the Non-redundant protein sequence (nr) database limiting the search to Mammalia.

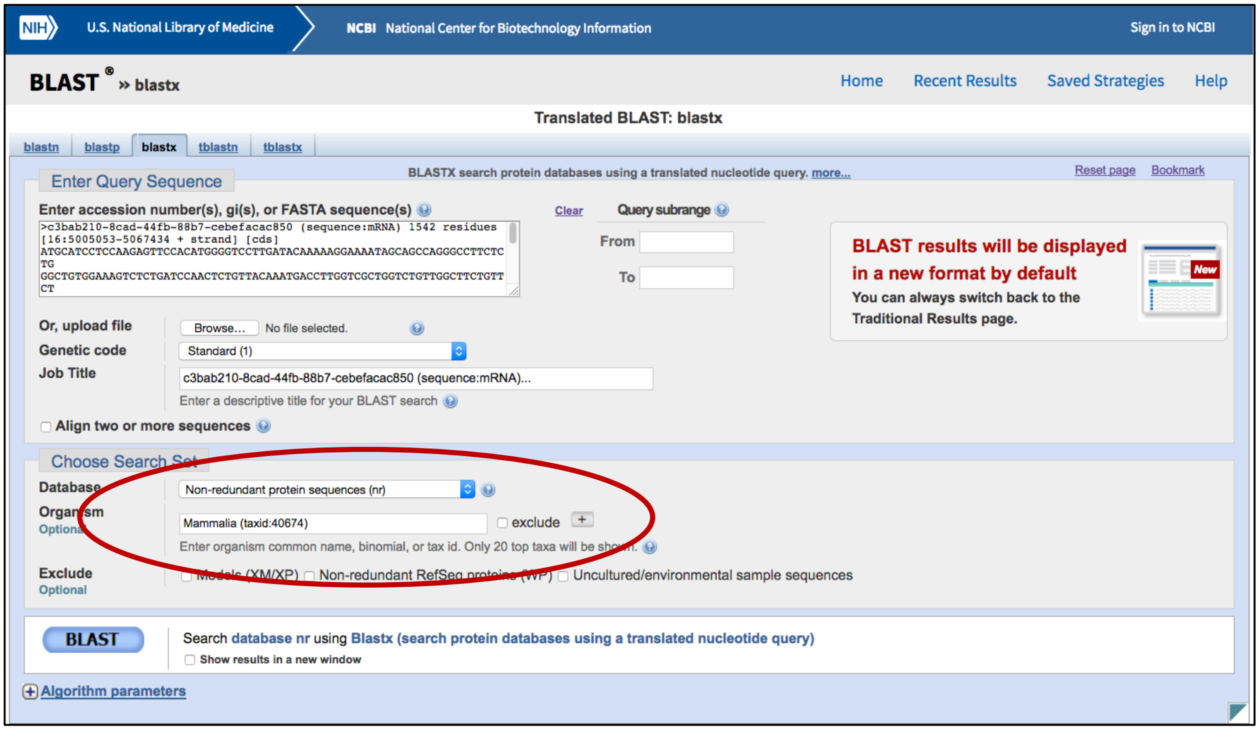

The BLASTX results show a good alignment with a protein in Texas white-tailed deer (albeit a “low quality protein”) (Fig. S56). The gaps shown in the alignment are not in the region of the non-canonical splice sites. By adding the lengths of the exons using the information about the SRR571259.32169 alignment (e.g. Fig. S54) we determined that the non-canonical splice site occurs around query positions 924. The alignment to the homologous deer protein is very good in that region. This evidence further validates the transcript with the non-canonical splice sites.

Figure S56. BLASTX alignment of the coding sequence of XM_002693872.6-00005 to a protein in the Non-redundant protein sequence (nr) database.

LOW QUALITY PROTEIN: C4b-binding protein alpha chain-like [Odocoileus virginianus texanus]

Sequence ID: XP_020770159.1 **Length:** 752 **Number of Matches:** 2

Range 1: 1 to 499 GenPept Graphics ▼ Next Match ▲ Previous Match

Related Information

Gene - associated gene details

Genome Data Viewer - aligned genomic context

Score	Expect	Method	Identities	Positives	Gaps	Frame
883 bits(2281)	0.0	Compositional matrix adjust.	428/500(86%)	454/500(90%)	9/500(1%)	+1

```
Query  1     MHPPRVPHGVLDTKRKIAARAFSGLWKVSDPTLLQMTLVAGLLASVLGDCGPPPDLQFAS  180
             MHPPRVPH  LDTKRK+ AR FSGLWKVSDPTLLQMTLVAGLLASVLGDCGPPPDLQFAS
Sbjct  1     MHPPRVPHAALDTKRKMVARPFSGLWKVSDPTLLQMTLVAGLLASVLGDCGPPPDLQFAS  60

Query  181   PDNKLNNKDFKTGTTLKYTCLPGYSR--------IKPSSVTCNDRGSWDYRVFCAKKQCR  336
             P+NKL+NKDFKTGTTLKYTCLPGYSR           SS+  N    +  ++FC  KQCR
Sbjct  61    PNNKLDNKDFKTGTTLKYTCLPGYSRXILXFKDYFDESSLQ-NXHYLYLEKIFCVYKQCR  119

Query  337   NLGDLPNGKIEVKTDFLFGSTVEFSCSEGYFLIGSPTSHCEIQDKGVDWSDPLPQCIIVK  516
             NLGDLPNGK+EVKTDFLFGST+EFSCSEGY L+GS TSHCEIQDKGVDWSDPLPQCII K
Sbjct  120   NLGDLPNGKVEVKTDFLFGSTIEFSCSEGYILVGSATSHCEIQDKGVDWSDPLPQCIIAK  179

Query  517   CKPPPTISNGKHNGGDEDFYTYGSSVTYSCDPDFSMLGKASISCRVENKTIGVWYPSPPT  696
             C+PPP ISNG+HNGGDEDFYTYGSSVTYSCDPDFSMLG+ASISCRVENKTIGVWYP+PPT
Sbjct  180   CEPPPAISNGRHNGGDEDFYTYGSSVTYSCDPDFSMLGQASISCRVENKTIGVWYPNPPT  239

Query  697   CKNIVCHRPRVRNGIILSGFGPIYHYKDSILFSCKKGYILNGNNLIHCDANNEWHPSPPT  876
             CKNIVCH PRVRNG ILSGFGPIYHYKDSILFSCKKGYIL+G++LI CDA+N+WHPSPPT
Sbjct  240   CKNIVCHPPRVRNGNILSGFGPIYHYKDSILFSCKKGYILSGDSLIRCDADNKWHPSPPT  299

Query  877   CELNSCIGLPDIPHAVWDGKDNKLSNQEVFEVGTELKYQCKFGYRHILDEPLTLTCQNNF  1056
             CELNSCIGLPDIPHAVWDG+DNKLSNQEVFEVGTELKYQCKFGYR I DEPLTLTC  NF
Sbjct  300   CELNSCIGLPDIPHAVWDGRDNKLSNQEVFEVGTELKYQCKFGYRPIPDEPLTLTCVENF  359

Query  1057  TWTTSKGCEYIVCHPPQVPNGNILSGFGPMYNFNDSVLFSCEKGYILNGSNLIHCGVDDE  1236
             TWT SKGCEYIVCHPPQVPNG ILSG GP+Y++ DSVLFSC+KGYILNG NLIHCG DDE
Sbjct  360   TWTPSKGCEYIVCHPPQVPNGIILSGVGPIYHYEDSVLFSCKKGYILNGRNLIHCGADDE  419

Query  1237  WHPSPPTCELITCLKPKVENGKLSVDKEKYVASENVTVQCNSGFSLVGPQSITCSENTTW  1416
             WHPS PTCELITCLKP+VENGKLSVDKEKYVASENVTV CN GFSLVGPQSITCSENTTW
Sbjct  420   WHPSLPTCELITCLKPEVENGKLSVDKEKYVASENVTVHCNPGFSLVGPQSITCSENTTW  479

Query  1417  HPTVPTCHWVNCTHVCPCMS  1476
             HPTVPTCHWVNCT VCPC+S
Sbjct  480   HPTVPTCHWVNCTIVCPCVS  499
```

In summary, we have determined that the RefSeq transcript model is correct and have added two transcript isoforms. Isoform XM_002693872.6-00004, which we did not further investigate, had an extra exon in the 5’ UTR, but the coding sequence was the same as XM_002693872.6-00001 (the original RefSeq transcript). We investigated the alternative exon structure of transcript XM_002693872.6-00005 because it had non-canonical splice sites. Although we did not find RNA-seq support for the alternative exons, a BLASTX alignment supported this isoform.
